# Supplementary material for: Single allele loss-of-function mutations select and sculpt conditional cooperative networks in breast cancer
Source: Nat Commun. 2021 Sep 2;12:5238. doi: 10.1038/s41467-021-25467-w (PMC8413298; doi:10.1038/s41467-021-25467-w)
Supplement: Supplementary file 1 — Supplementary Information [file 41467_2021_25467_MOESM1_ESM.pdf]

Single allele loss-of-function mutations select and sculpt conditional cooperative networks in breast cancer

Schacter et al.

Supplementary Information

Supplementary Figure 1: Schematic illustration of large-scale Sleeping Beauty mammary cancer gene discovery screens with multiple GEMM

Supplementary Figure 2: Kaplan-Meier survival analysis for Sleeping Beauty cancer gene discovery screens in GEMM models of breast cancer

Supplementary Figure 3: Oncoprint analysis shows complete list of clonal gCIS from each GEMM-specific cohort

Supplementary Figure 4: Schematic shows SB insertion sites which are predicted to represent gain-of-function alleles

Supplementary Figure 5: Mammary Tumor Pathology Guide

Supplementary Figure 6. Histopathologic classification of SB-induced mammary tumors from each driver-specific cohort

Supplementary Figure 7. Oncoprint analysis shows complete list of clonal gCIS from each histopathology-specific cohort

Supplementary Table 1: Reagents and Resources

Supplementary References

Supplementary Figure 1

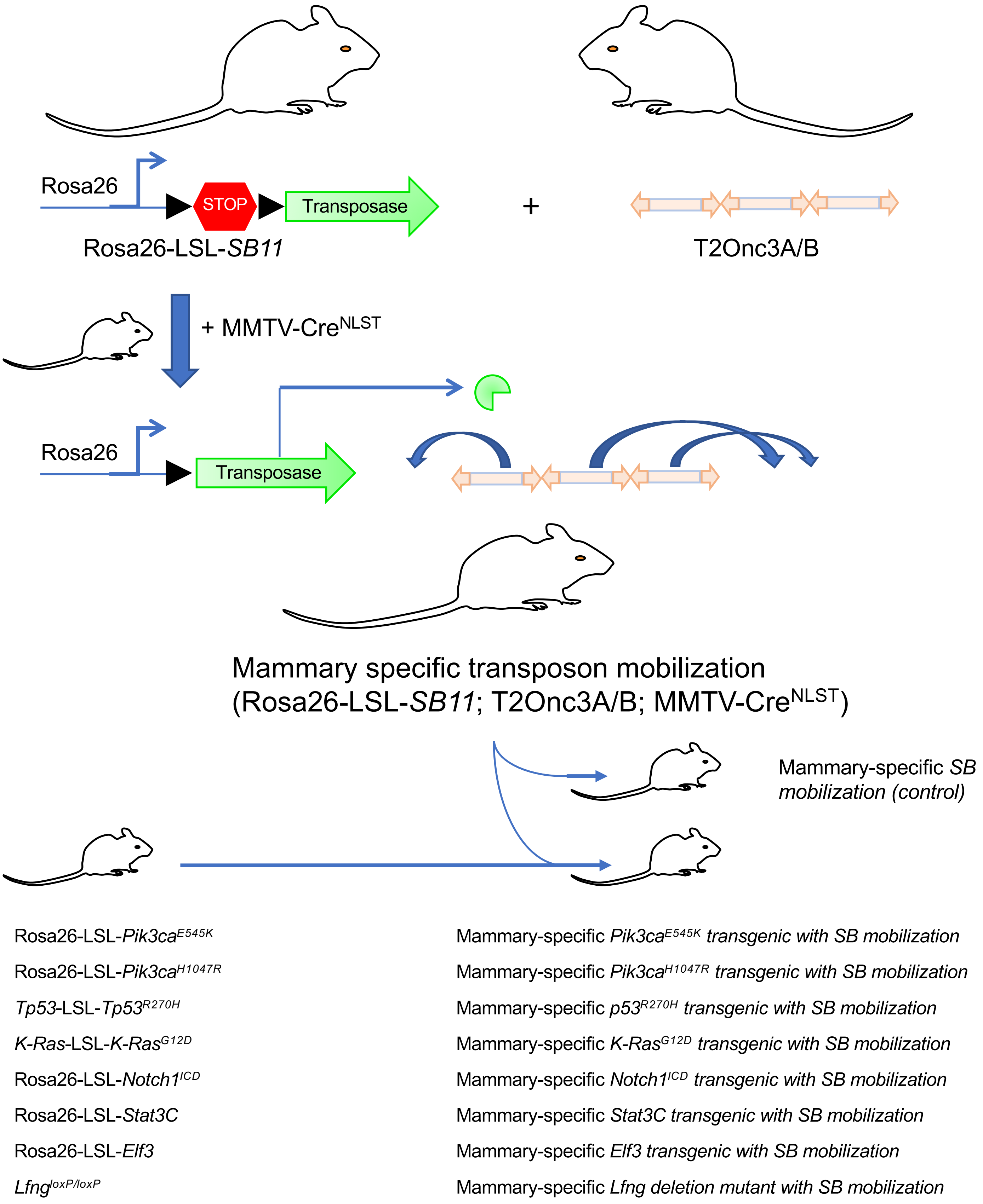

Supplementary Figure 1: Schematic illustration of large-scale Sleeping Beauty mammary cancer gene discovery screens with multiple GEMM

Supplementary Figure 2

Control

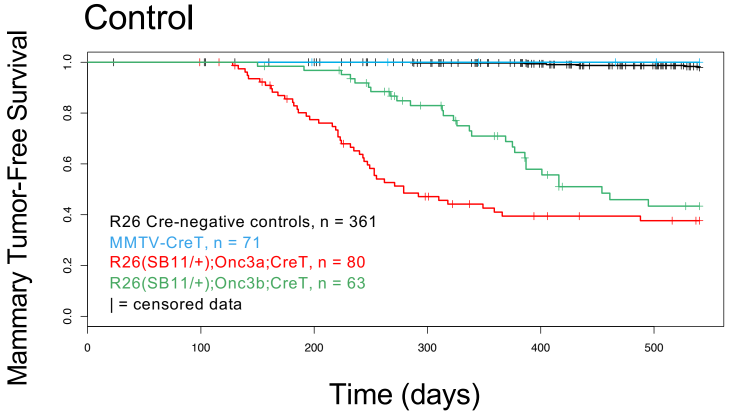

Cohort 1	Cohort 2	p-value
R26 Cre-negative controls	MMTV-Cre^{NLST}	0.0182
R26 Cre-negative controls	R26$^{SB11/+}$;Onc3a	0
R26 Cre-negative controls	R26$^{SB11/+}$;Onc3b	0
MMTV-CreT	R26$^{SB11/+}$;Onc3a	0
MMTV-CreT	R26$^{SB11/+}$;Onc3b	0
R26$^{SB11/+}$;Onc3a	R26$^{SB11/+}$;Onc3b	0.0145

Pik3ca^{E545K}

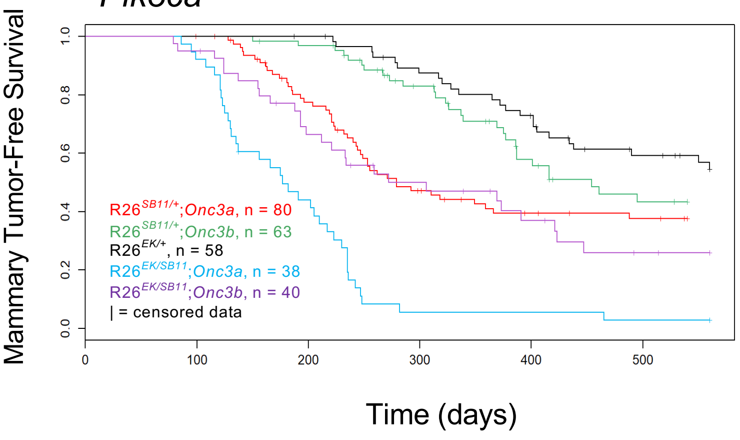

Cohort 1	Cohort 2	p-value
R26$^{SB11/+}$;Onc3a	R26$^{EK/+}$	0.00032
R26$^{SB11/+}$;Onc3b	R26$^{EK/+}$	0.106
R26$^{EK/+}$	R26$^{EK/SB11}$;Onc3a	0
R26$^{EK/+}$	R26$^{EK/SB11}$;Onc3b	8.25x10^{-5}
R26$^{SB11/+}$;Onc3a	R26$^{EK/SB11}$;Onc3a	6.29x10^{-10}
R26$^{SB11/+}$;Onc3b	R26$^{EK/SB11}$;Onc3b	0.00392

Pik3ca^{H1047R}

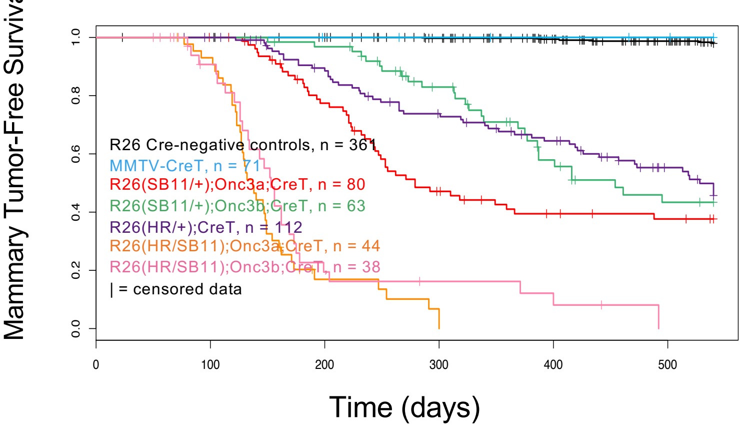

Cohort 1	Cohort 2	p-value
R26$^{SB11/+}$;Onc3a	R26$^{HR/+}$	0.0225
R26$^{SB11/+}$;Onc3b	R26$^{HR/+}$	0.977
R26$^{HR/+}$	R26$^{HR/SB11}$;Onc3a	0
R26$^{HR/+}$	R26$^{HR/SB11}$;Onc3b	0
R26$^{SB11/+}$;Onc3a	R26$^{HR/SB11}$;Onc3a	1.11x10^{-16}
R26$^{SB11/+}$;Onc3b	R26$^{HR/SB11}$;Onc3b	4.44x10^{-15}

p53^{R270H}

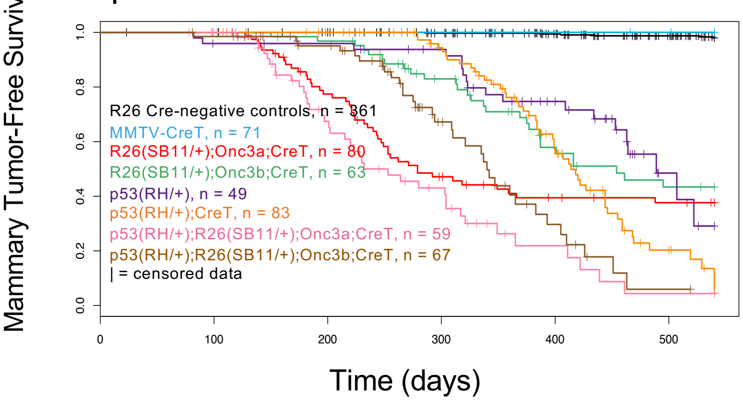

Cohort 1	Cohort 2	p-value
p53$^{RH/+}$ Cre negative	p53$^{RH/+}$	0.00223
R26$^{SB11/+}$;Onc3a	p53$^{RH/+}$	0.497
R26$^{SB11/+}$;Onc3b	p53$^{RH/+}$	0.0535
p53$^{RH/+}$	p53$^{RH/+}$;R26$^{SB11/+}$;Onc3a	8.69x10^{-8}
p53$^{RH/+}$	p53$^{RH/+}$;R26$^{SB11/+}$;Onc3b	0.000737
R26$^{SB11/+}$;Onc3a	p53$^{RH/+}$;R26$^{SB11/+}$;Onc3a	0.0124
R26$^{SB11/+}$;Onc3b	p53$^{RH/+}$;R26$^{SB11/+}$;Onc3b	0.00051

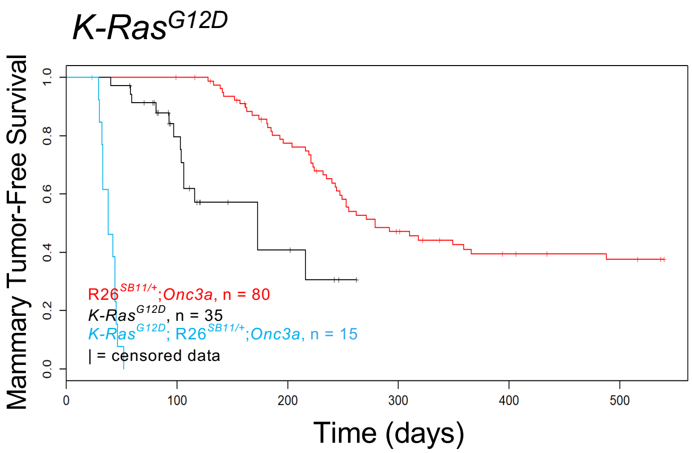

Cohort 1	Cohort 2	p-value
$R26^{SB11/+}$;Onc3a	$K\text{-}Ras^{G12D}$	$8.87x10^{-6}$
$K\text{-}Ras^{G12D}$	$K\text{-}Ras^{G12D}$;$R26^{SB11/+}$;Onc3a	$2.44x10^{-15}$
$R26^{SB11/+}$;Onc3a	$K\text{-}Ras^{G12D}$;$R26^{SB11/+}$;Onc3a	0

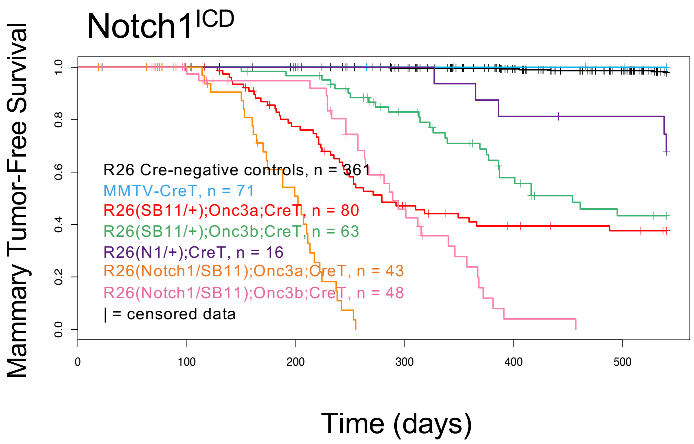

Cohort 1	Cohort 2	p-value
$R26^{SB11/+}$;Onc3a	$R26^{N1/+}$	0.00947
$R26^{SB11/+}$;Onc3b	$R26^{HR/+}$	0.0628
$R26^{N1/+}$	$R26^{N1/SB11}$;Onc3a	$2.06x10^{-10}$
$R26^{N1/+}$	$R26^{N1/SB11}$;Onc3b	$2.52x10^{-8}$
$R26^{SB11/+}$;Onc3a	$R26^{N1/SB11}$;Onc3a	$1.27x10^{-9}$
$R26^{SB11/+}$;Onc3b	$R26^{N1/SB11}$;Onc3b	$8.98x10^{-9}$

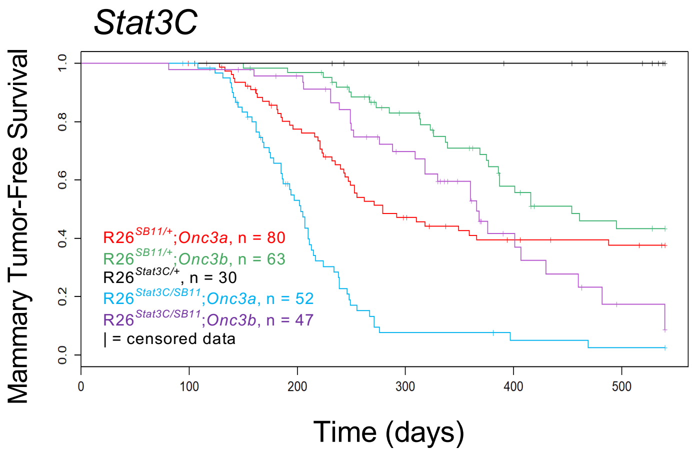

Cohort 1	Cohort 2	p-value
$R26^{SB11/+}$;Onc3a	$R26^{Stat3C/+}$	$2.92x10^{-7}$
$R26^{SB11/+}$;Onc3b	$R26^{Stat3C/+}$	$5.05x10^{-6}$
$R26^{Stat3C/+}$	$R26^{Stat3C/SB11}$;Onc3a	$3.33x10^{-16}$
$R26^{Stat3C/+}$	$R26^{Stat3C/SB11}$;Onc3b	$3.98x10^{-10}$
$R26^{SB11/+}$;Onc3a	$R26^{Stat3C/SB11}$;Onc3a	$1.61x10^{-8}$
$R26^{SB11/+}$;Onc3b	$R26^{Stat3C/SB11}$;Onc3b	0.00776

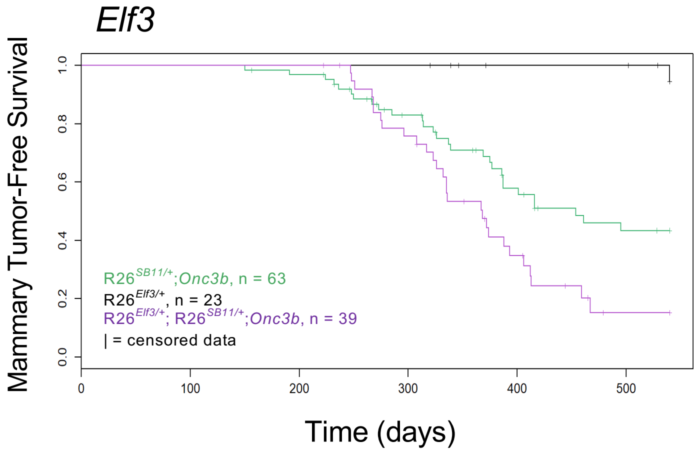

Cohort 1	Cohort 2	p-value
$R26^{SB11/+}$;Onc3b	$R26^{Elf3/+}$	$8.93x10^{-5}$
$R26^{Elf3/+}$	$R26^{Elf3/SB11}$;Onc3b	$9.73x10^{-9}$
$R26^{SB11/+}$;Onc3b	$R26^{Elf3/SB11}$;Onc3b	0.0118

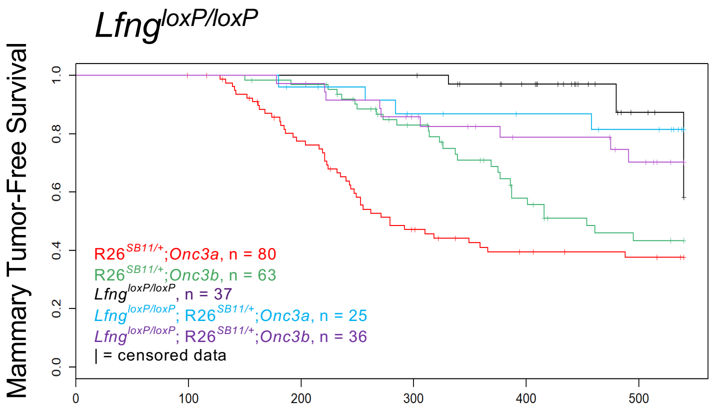

Cohort 1	Cohort 2	p-value
R26$^{SB11/+}$;Onc3a	Lfng$^{loxP/loxP}$	6.32×10^{-7}
R26$^{SB11/+}$;Onc3b	Lfng$^{loxP/loxP}$	0.000191
Lfng$^{loxP/loxP}$	Lfng$^{loxP/loxP}$;R26$^{SB11/+}$;Onc3a	0.596
Lfng$^{loxP/loxP}$	Lfng$^{loxP/loxP}$;R26$^{SB11/+}$;Onc3b	0.127
R26$^{SB11/+}$;Onc3a	Lfng$^{loxP/loxP}$;R26$^{SB11/+}$;Onc3b	0.000534
R26$^{SB11/+}$;Onc3b	Lfng$^{loxP/loxP}$;R26$^{SB11/+}$;Onc3b	0.0413

Supplementary Figure 2: Kaplan-Meier survival analysis for Sleeping Beauty cancer gene discovery screens in GEMM models of breast cancer

Kaplan-Meier survival analysis shows cooperation between initiating events in each GEMM, except *Lfng*$^{loxP/loxP}$, and SB mutagenesis. All mice are positive for MMTV-Cre^{NLST}, except for Cre-negative controls as noted. Survival statistics were calculated as non-parametric log rank p-values.

Control

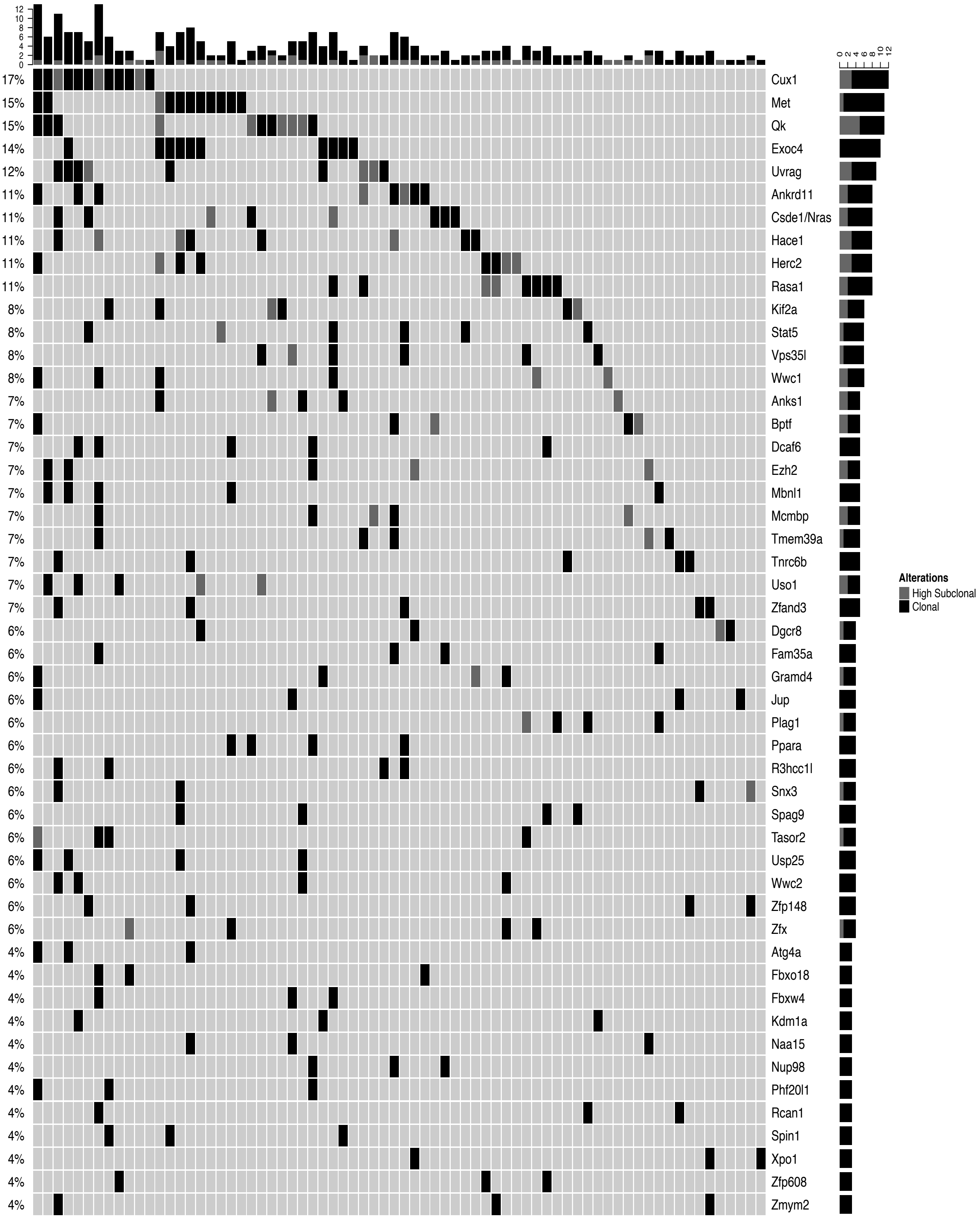

Cux1 17%
Met 15%
Qk 15%
Exoc4 14%
Uvrag 12%
Ankrd11 11%
Csde1/Nras 11%
Hace1 11%
Herc2 11%
Rasa1 11%
Kif2a 8%
Stat5 8%
Vps35l 8%
Wwc1 8%
Anks1 7%
Bptf 7%
Dcaf6 7%
Ezh2 7%
Mbnl1 7%
Mcmbp 7%
Tmem39a 7%
Tnrc6b 7%
Uso1 7%
Zfand3 7%
Dgcr8 6%
Fam35a 6%
Gramd4 6%
Jup 6%
Plag1 6%
Ppara 6%
R3hcc1l 6%
Snx3 6%
Spag9 6%
Tasor2 6%
Usp25 6%
Wwc2 6%
Zfp148 6%
Zfx 6%
Atg4a 4%
Fbxo18 4%
Fbxw4 4%
Kdm1a 4%
Naa15 4%
Nup98 4%
Phf20l1 4%
Rcan1 4%
Spin1 4%
Xpo1 4%
Zfp608 4%
Zmym2 4%
Alterations
High Subclonal
Clonal

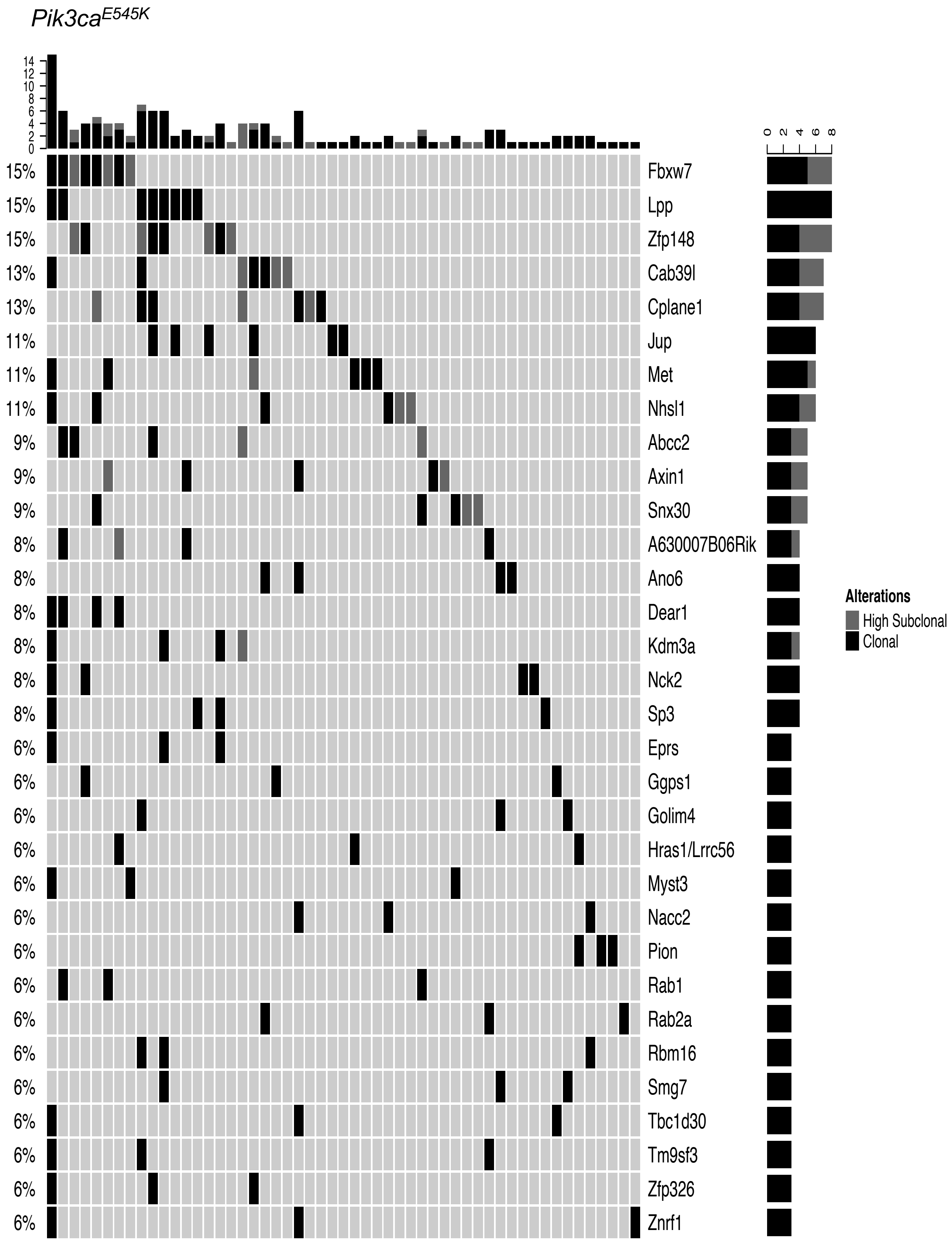

Pik3caE545K
14
12
10
8
6
4
2
0
15% Fbxw7
15% Lpp
15% Zfp148
13% Cab39l
13% Cplane1
11% Jup
11% Met
11% Nhsl1
9% Abcc2
9% Axin1
9% Snx30
8% A630007B06Rik
8% Ano6
8% Dear1
8% Kdm3a
8% Nck2
8% Sp3
6% Eprs
6% Ggps1
6% Golim4
6% Hras1/Lrrc56
6% Myst3
6% Nacc2
6% Pion
6% Rab1
6% Rab2a
6% Rbm16
6% Smg7
6% Tbc1d30
6% Tm9sf3
6% Zfp326
6% Znrf1
0 2 4 6 8
Alterations
High Subclonal
Clonal

Pik3ca^{H1047R}

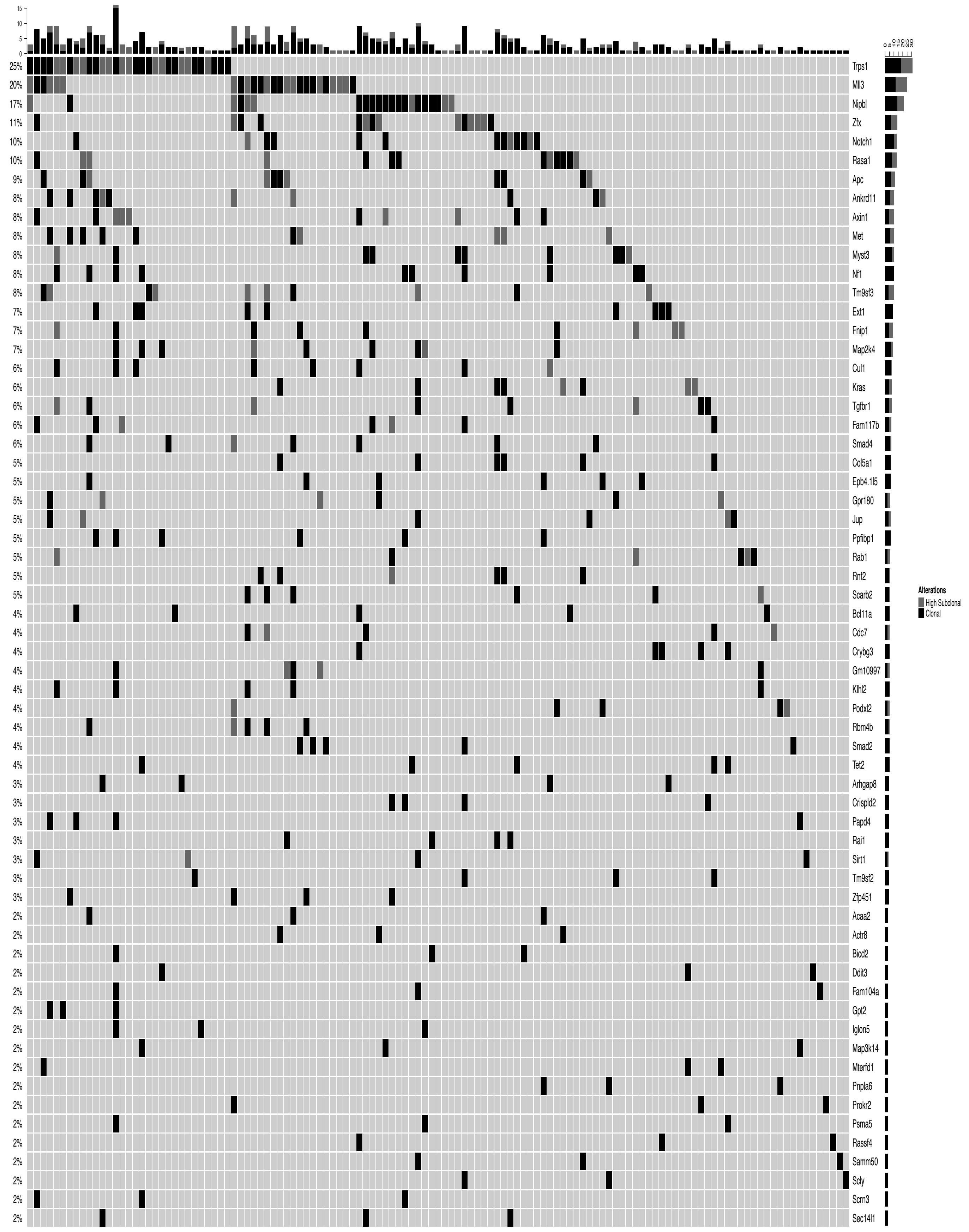

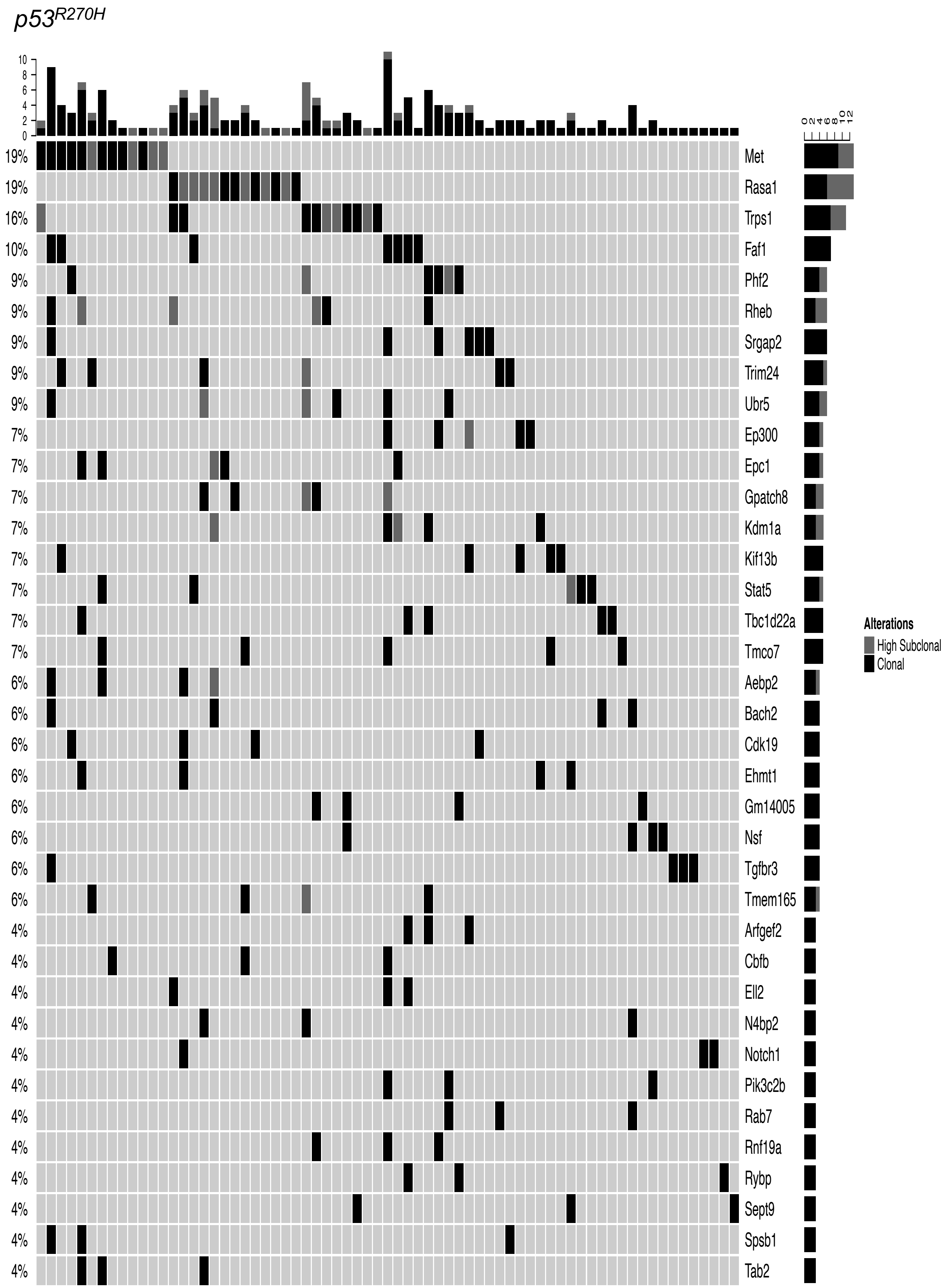

p53R270H
Met
Rasa1
Trps1
Faf1
Phf2
Rheb
Srgap2
Trim24
Ubr5
Ep300
Epc1
Gpatch8
Kdm1a
Kif13b
Stat5
Tbc1d22a
Tmco7
Aebp2
Bach2
Cdk19
Ehmt1
Gm14005
Nsf
Tgfbr3
Tmem165
Arfgef2
Cbfb
Ell2
N4bp2
Notch1
Pik3c2b
Rab7
Rnf19a
Rybp
Sept9
Spsb1
Tab2
19%
19%
16%
10%
9%
9%
9%
9%
9%
7%
7%
7%
7%
7%
7%
7%
7%
6%
6%
6%
6%
6%
6%
6%
6%
4%
4%
4%
4%
4%
4%
4%
4%
4%
4%
4%
4%
Alterations
High Subclonal
Clonal

Kras^G12D

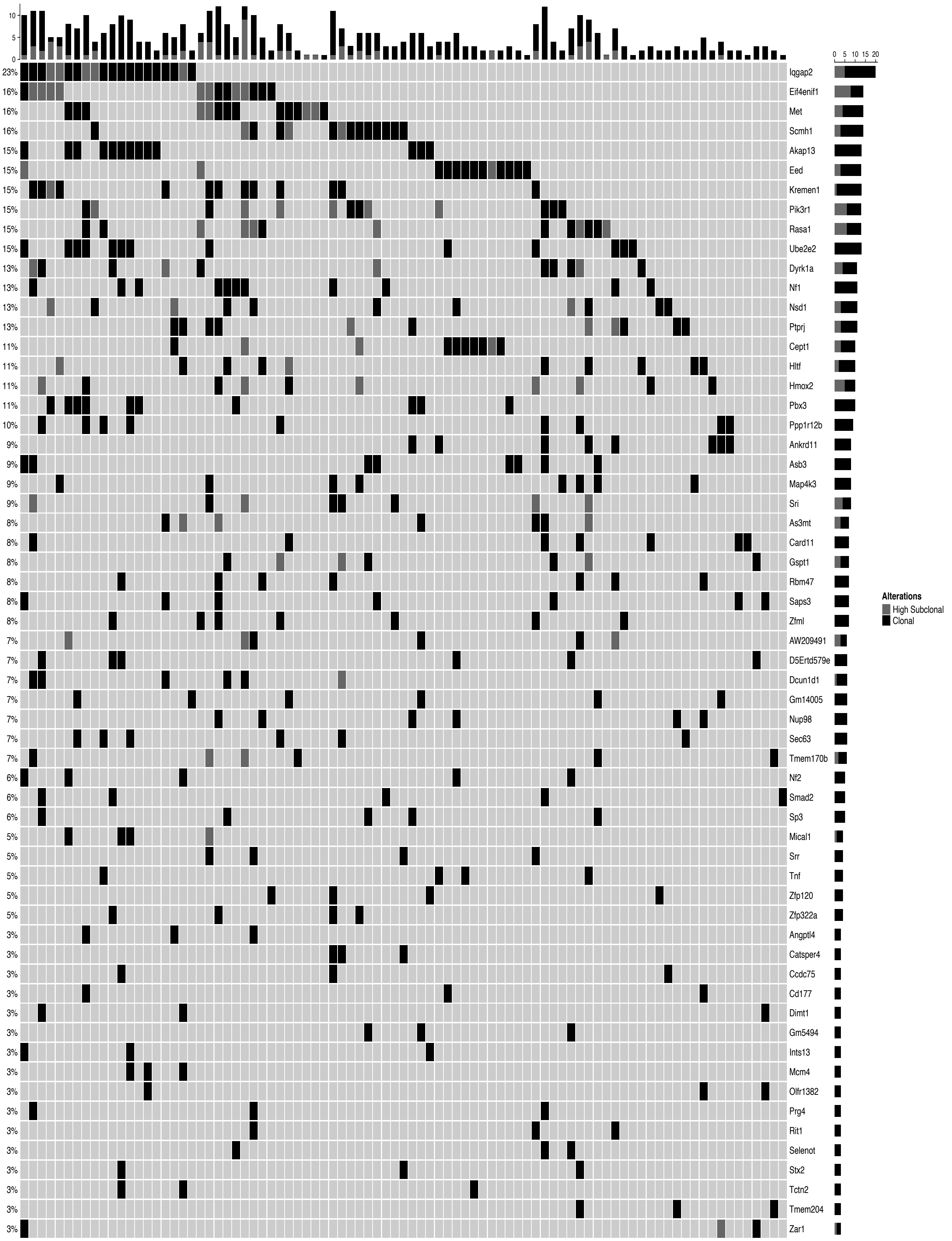
0
5
10
0 5 10 15 20
23% Iqgap2
16% Eif4enif1
16% Met
16% Scmh1
15% Akap13
15% Eed
15% Kremen1
15% Pik3r1
15% Rasa1
15% Ube2e2
13% Dyrk1a
13% Nf1
13% Nsd1
13% Ptprj
11% Cept1
11% Hltf
11% Hmox2
11% Pbx3
10% Ppp1r12b
9% Ankrd11
9% Asb3
9% Map4k3
9% Sri
8% As3mt
8% Card11
8% Gspt1
8% Rbm47
8% Saps3
8% Zfml
7% AW209491
7% D5Ertd579e
7% Dcun1d1
7% Gm14005
7% Nup98
7% Sec63
7% Tmem170b
6% Nf2
6% Smad2
6% Sp3
5% Mical1
5% Srr
5% Tnf
5% Zfp120
5% Zfp322a
3% Angptl4
3% Catsper4
3% Ccdc75
3% Cd177
3% Dimt1
3% Gm5494
3% Ints13
3% Mcm4
3% Olfr1382
3% Prg4
3% Rit1
3% Selenot
3% Stx2
3% Tctn2
3% Tmem204
3% Zar1
Alterations
High Subclonal
Clonal

Notch1^{ICD}

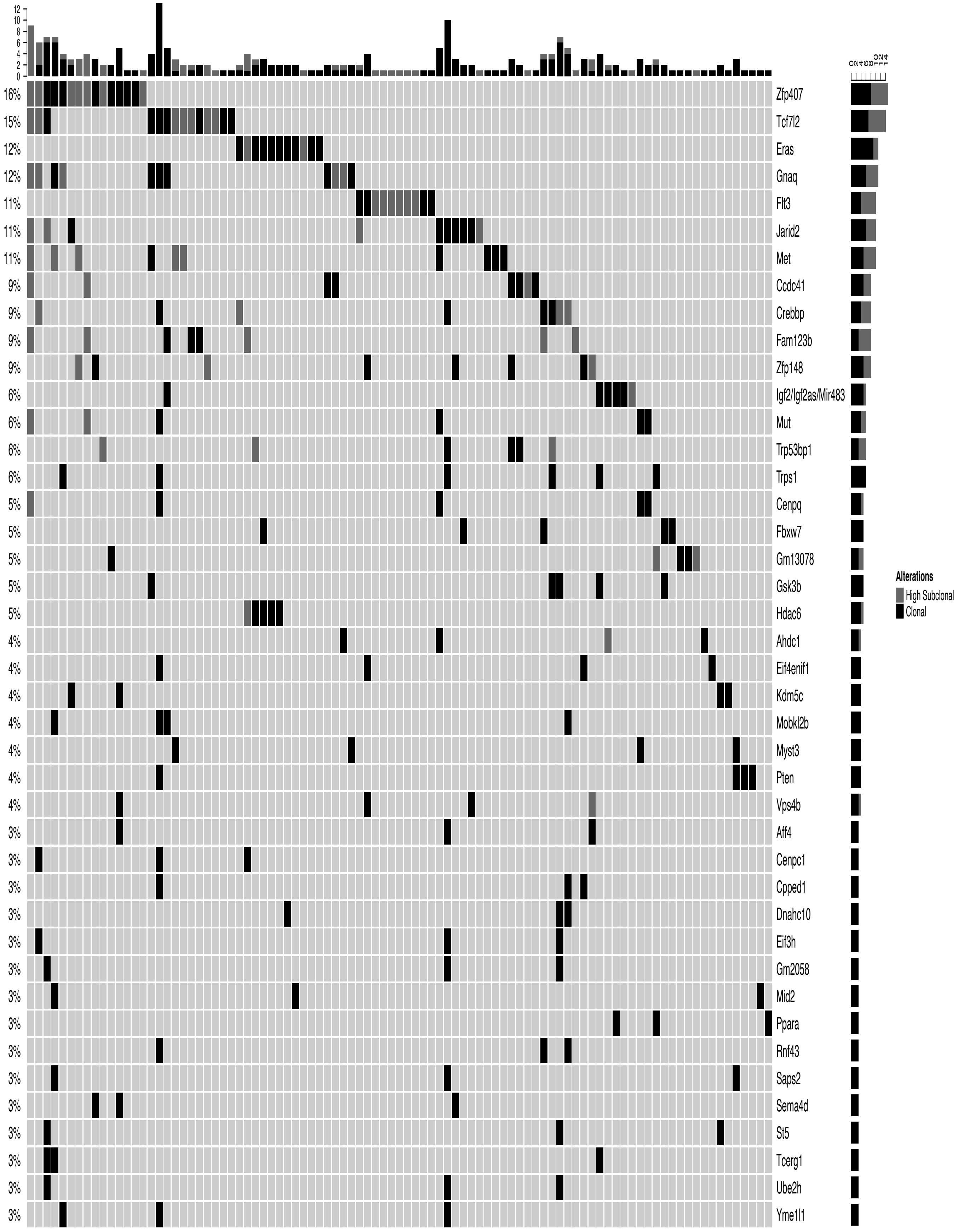

0
2
4
6
8
10
12
16%
15%
12%
12%
11%
11%
11%
9%
9%
9%
9%
6%
6%
6%
6%
5%
5%
5%
5%
5%
4%
4%
4%
4%
4%
4%
4%
3%
3%
3%
3%
3%
3%
3%
3%
3%
3%
3%
3%
3%
3%
Zfp407
Tcf7l2
Eras
Gnaq
Flt3
Jarid2
Met
Ccdc41
Crebbp
Fam123b
Zfp148
Igf2/Igf2as/Mir483
Mut
Trp53bp1
Trps1
Cenpq
Fbxw7
Gm13078
Gsk3b
Hdac6
Ahdc1
Eif4enif1
Kdm5c
Mobkl2b
Myst3
Pten
Vps4b
Aff4
Cenpc1
Cpped1
Dnahc10
Eif3h
Gm2058
Mid2
Ppara
Rnf43
Saps2
Sema4d
St5
Tcerg1
Ube2h
Yme1l1
Alterations
High Subclonal
Clonal

Stat3c

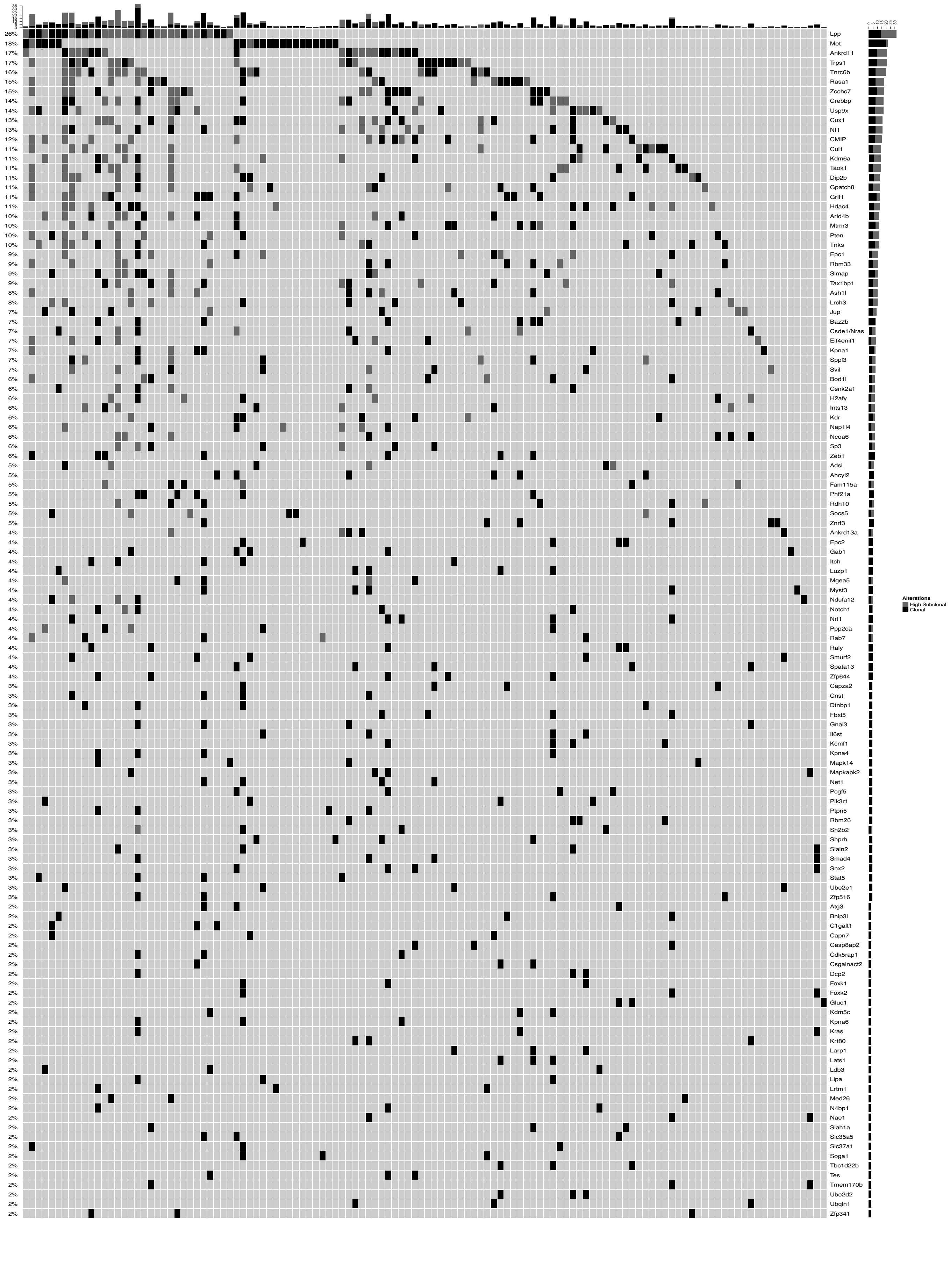

26% Lpp
18% Met
17% Ankrd11
17% Trps1
16% Tnrc6b
15% Rasa1
15% Zcchc7
14% Crebbp
14% Usp9x
13% Cux1
13% Nf1
12% CMIP
11% Cul1
11% Kdm6a
11% Taok1
11% Dip2b
11% Gpatch8
11% Grlf1
11% Hdac4
10% Arid4b
10% Mtmr3
10% Pten
10% Tnks
9% Epc1
9% Rbm33
9% Slmap
9% Tax1bp1
8% Ash1l
8% Lrch3
7% Jup
7% Baz2b
7% Csde1/Nras
7% Eif4enif1
7% Kpna1
7% Sppl3
7% Svil
6% Bod1l
6% Csnk2a1
6% H2afy
6% Ints13
6% Kdr
6% Nap1l4
6% Ncoa6
6% Sp3
6% Zeb1
5% Adsl
5% Ahcyl2
5% Fam115a
5% Phf21a
5% Rdh10
5% Socs5
5% Znrf3
4% Ankrd13a
4% Epc2
4% Gab1
4% Itch
4% Luzp1
4% Mgea5
4% Myst3
4% Ndufa12
4% Notch1
4% Nrf1
4% Ppp2ca
4% Rab7
4% Raly
4% Smurf2
4% Spata13
4% Zfp644
3% Capza2
3% Cnst
3% Dtnbp1
3% Fbxl5
3% Gnai3
3% Il6st
3% Kcmf1
3% Kpna4
3% Mapk14
3% Mapkapk2
3% Net1
3% Pcgf5
3% Pik3r1
3% Ptpn5
3% Rbm26
3% Sh2b2
3% Shprh
3% Slain2
3% Smad4
3% Snx2
3% Stat5
3% Ube2e1
3% Zfp516
2% Atg3
2% Bnip3l
2% C1galt1
2% Capn7
2% Casp8ap2
2% Cdk5rap1
2% Csgalnact2
2% Dcp2
2% Foxk1
2% Foxk2
2% Glud1
2% Kdm5c
2% Kpna6
2% Kras
2% Krt80
2% Larp1
2% Lats1
2% Ldb3
2% Lipa
2% Lrtm1
2% Med26
2% N4bp1
2% Nae1
2% Siah1a
2% Slc35a5
2% Slc37a1
2% Soga1
2% Tbc1d22b
2% Tes
2% Tmem170b
2% Ube2d2
2% Ubqln1
2% Zfp341
Alterations
High Subclonal
Clonal

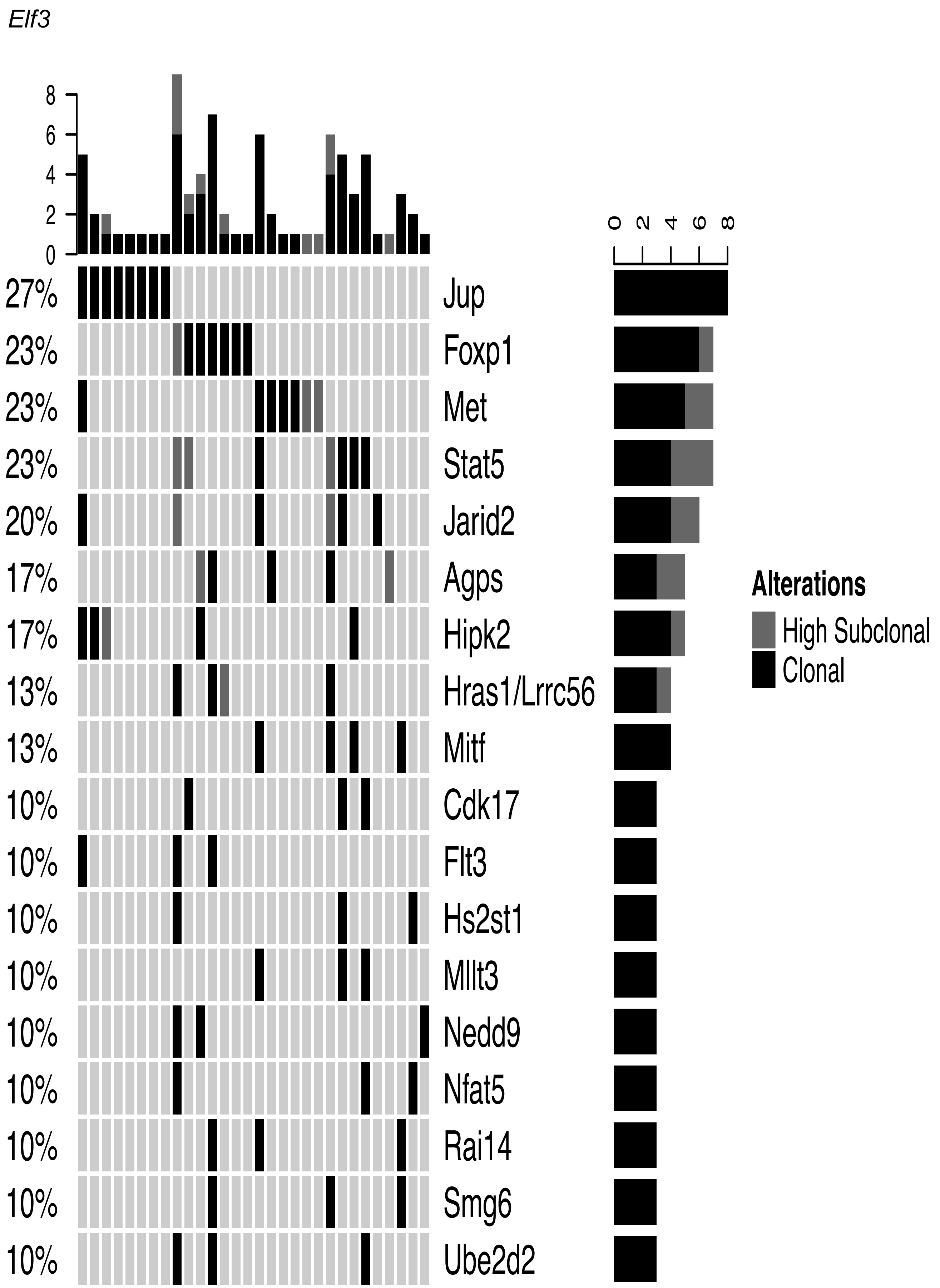

Elf3
8
6
4
2
0
27% Jup
23% Foxp1
23% Met
23% Stat5
20% Jarid2
17% Agps
17% Hipk2
13% Hras1/Lrrc56
13% Mitf
10% Cdk17
10% Flt3
10% Hs2st1
10% Mllt3
10% Nedd9
10% Nfat5
10% Rai14
10% Smg6
10% Ube2d2
Alterations
High Subclonal
Clonal

Lfng$^{-/-}$

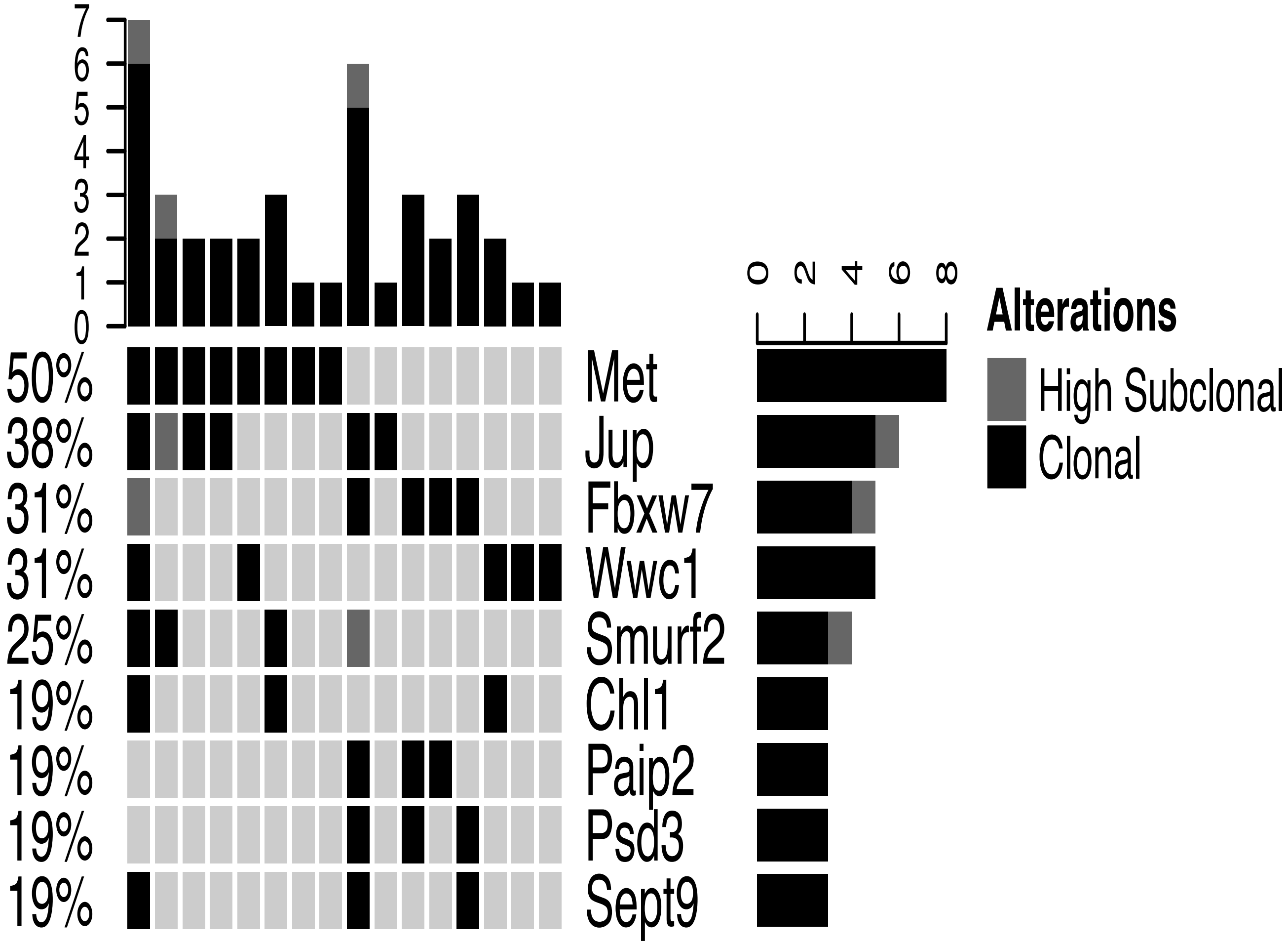

7
6
5
4
3
2
1
0
50%
38%
31%
31%
25%
19%
19%
19%
19%
Met
Jup
Fbxw7
Wwc1
Smurf2
Chl1
Paip2
Psd3
Sept9
0
2
4
6
8
Alterations
High Subclonal
Clonal

Large Cohort

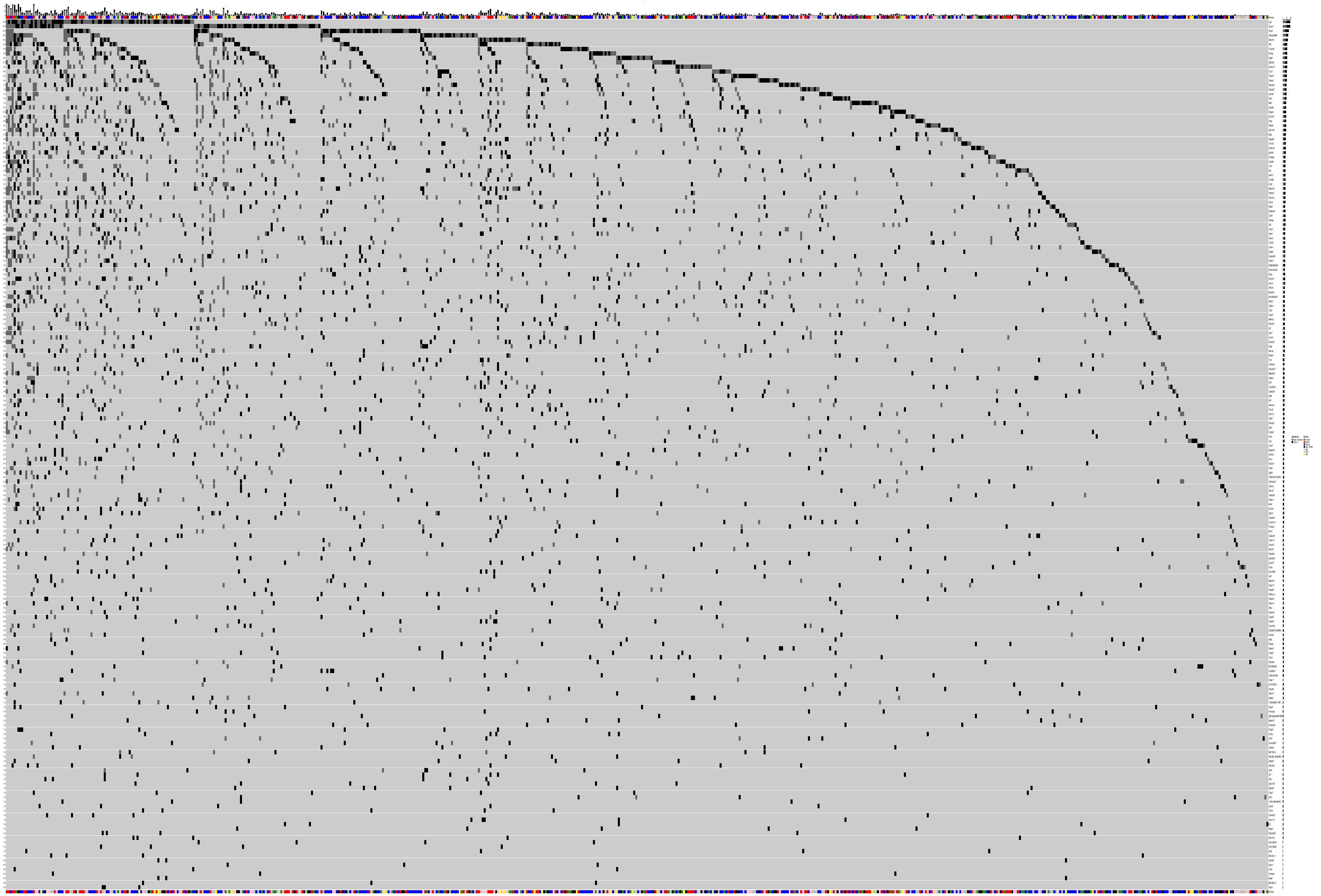

Supplementary Figure 3: Oncoprint analysis shows complete list of clonal gCIS from each GEMM-specific cohort

List of Clonal gCIS (with subclonal tumors added) from each GEMM SB screen. Note, the percentage of tumors with each gene targeted by SB is shown on the y-axis to the left for each bar graph, the number of tumors with clonal vs. subclonal SB targeting by SB is shown on the y-axis to the right, whereas the number of identified gCIS in each tumor is shown on the x-axis above each bar graph. Note, for example, a high-frequency gCIS identified in our Rosa26-LSL-*Pik3ca*^{E545K}-SB cohort was *Fbxw7*. This gene had SB insertions in 15% of tumors from the cohort (left side y-axis). Also, as shown on the right hand y-axis, clonal insertions were identified in 5/8 tumors from the cohort with insertions in the gene, whereas the remaining 3/8 had subclonal inserts. Finally, the tumor depicted in the most left hand column from the same cohort (Rosa26-LSL-*Pik3ca*^{E545K}-SB) had 14 different gCIS insertions, all of which scored as clonal.

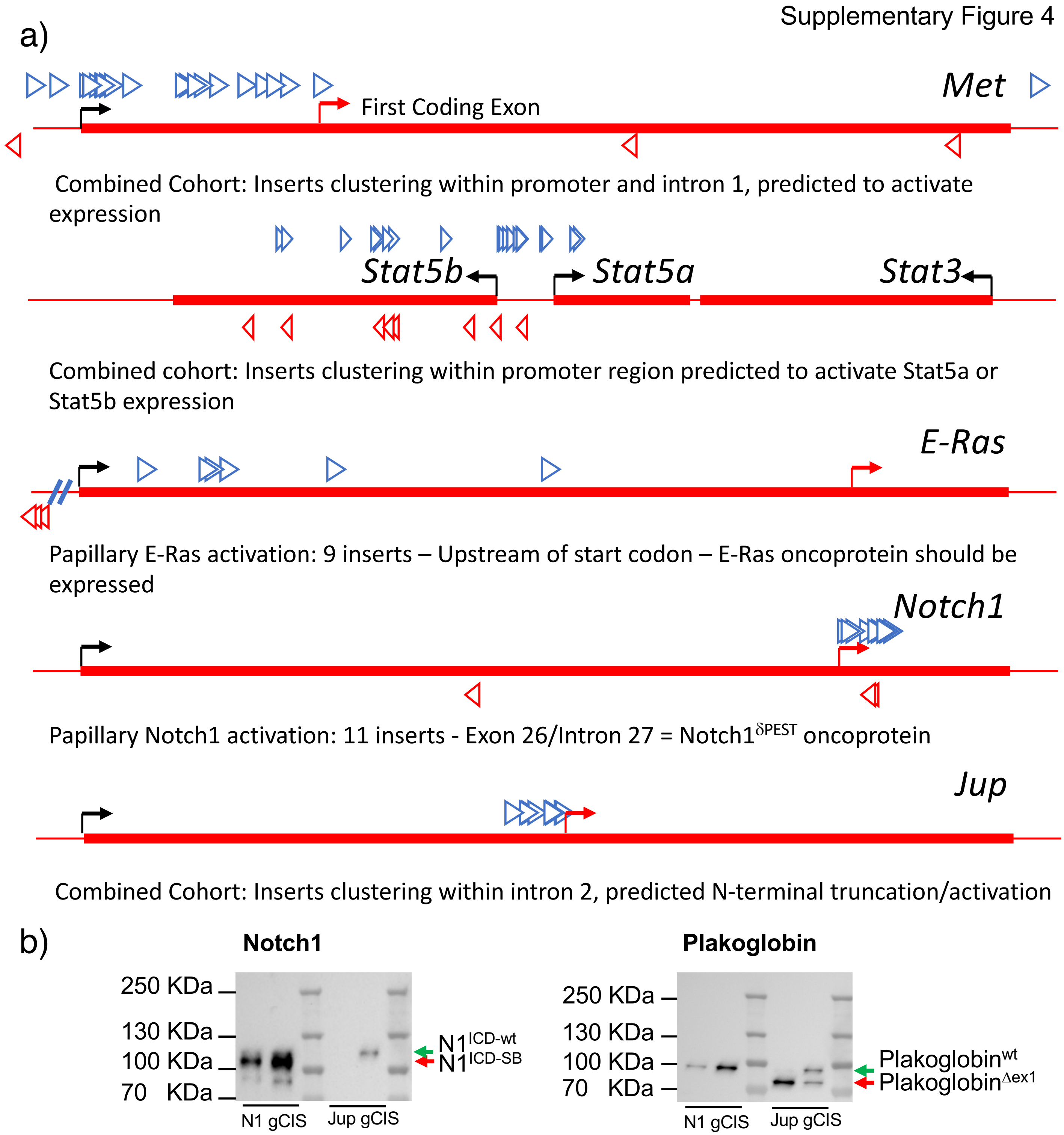

Supplementary Figure 4: Schematic shows SB insertion sites which are predicted to represent gain-of-function alleles. (a) gCIS with predicted gain-of-function insertions. Black arrows show transcriptional start site for the gene of interest. Red arrows show predicted start codon for most SB-induced alleles. (b) Western analysis for Notch1 and Plakoglobin proteins in tumors with SB insertions in either *Notch1* or *Jup* (the gene for Plakoglobin). Seen are N-terminal truncation fragments, which are predicted on the basis on SB insertion within introns and oriented in the same direction as the gene itself ($N1^{ICD-SB}$ = SB-insertion induced $Notch1^{ICD}$ protein fragment and $Plakoglobin^{□ex1}$ = SB-induced Plakoglobin protein fragment). Images shown are two biological replicates for tumors with Notch 1 insertions and two biological replicates for tumors with Jup insertions. Raw data for these western blots in Source data file.

Supplementary Figure 5

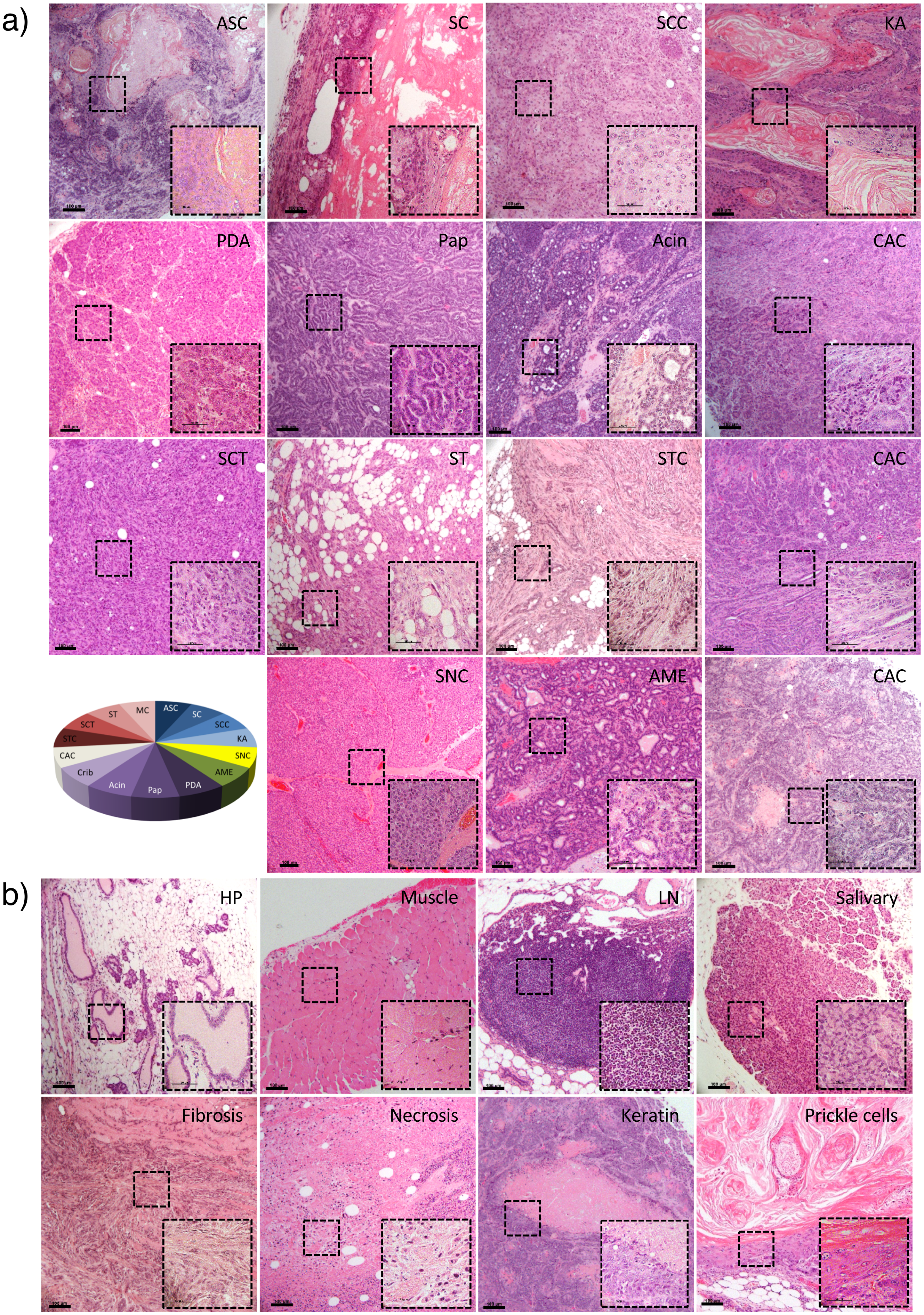

a) Tumor type
ASC – Adenosquamous carcinoma: mixture of glandular and squamous elements; glandular regions are derived from mammary epithelial cells and have poorly differentiated, acinar, or papillary features. Squamous regions composed of fat light pink keratin-producing cells (Prickle cells). Regions of necrosis, evident in this section, are common. **SC** – Squamous cyst: similar features to ASC but limited glandular and squamous cells present; mostly composed of keratin. **SCC** – Squamous cell carcinoma: Mammary tumor predominantly composed of keratin-producing cells (under high magnification these cells are identified as Prickle cells) with small numbers of keratin pockets. **KA** – Keratoacanthoma: a form of SCC with large keratin deposits surrounded by stratified squamous cells with little to no mammary epithelium. **PDA** – Poorly differentiated adenocarcinoma: solid sheet of round or cuboidal cells with minimal cytoplasm, and no glandular structure. **Pap** – Papillary: poorly differentiated cells that form complex network of finger-like projections interspersed with varying degrees of fibrosis. **Acin** – Acinar: small clusters of cells (typically one layer) that surround a small central lumen. **CAC** – Complex adenocarcinoma: Heterogeneous tumor composed of two or more tumor types (i.e. squamous, spindle, adenocarcinoma, AME, SNC). **SCT** – Spindle cell tumor: mammary epithelial glandular structures replaced with sheets of long, narrow and tapered mesenchymal cells; generally tumor has defined borders. **ST** – Scirrhous tumor: invasive stellate-shaped tumor composed of spindle/mesenchymal cells with minimal glandular regions. **STC** – Scirrhous tubular carcinoma: invasive tumor with glandular regions (some retain mammary ductal structure) and extensive fibrosis with EMT. **SNC** – Solid nodular carcinoma: highly vascular tumor composed of uniform glandular cells with slight nuclear atypia. **AME** – Adenomyoepithelioma: differentiated tumor composed of glandular epithelium surrounded by myoepithelial cells, varying degree of epithelial-to-mesenchymal transition (EMT). **Pie chart** – tumor subtypes commonly observed in mouse models of breast cancer. Tumors are grouped according to morphological features and biomarker expression (blue: squamous, green: adenomyoepitheliomas, yellow: solid nodular carcinoma, purple: adenocarcinoma, red: spindle/metaplastic, white: complex tumors composed of two or more subtypes). Images shown are representative of at least 64, 32, 231, 23, 55, 4, 106, 5, 3, 7 and 8 mouse mammary tumors with ASC, SC, SCC, KA, PDA, Acinar, CAC, ST, STC, SNC and AME histology. SCT tumors were not identified in any experimental animals within SB cohort mice. However, the SCT tumor shown above is representative of 170 such tumors identified in the Egan lab (PMID: 30332649). Scale bars in each large panel = 100 μm. Inset images are expanded by 2.4 fold.

b) Features
HP – Hyperplasia: Abnormal cell growth resulting in multilayered luminal compartment. **Muscle** – Skeletal muscle: striated tissue formed from multiple bundles of cells; Dark pink striated and multinucleated structures usually found near mammary glands 3-5 (image representative of normal, healthy muscle). **LN** – Lymph nods are oval or kidney-shaped secondary lymphoid organs in which lymphocytes mature; LNs containing tiny dark purple nuclei (lymphocytes) surrounded by a fibrous capsule. **Salivary gland** - saliva-producing exocrine gland located bilaterally in the neck; raspberry-like structures that are often harvested with mammary glands 1-3. **Fibrosis**: An increase in fibrous connective tissue. **Necrosis**: Amorphous pink material with cellular debris. **Keratin**: Fibrous structural proteins secreted by squamous cells; deep pink swirls often surrounded by squamous cells.
Prickle cells: Keratin-producing cells, also known as spinous cells, normally found in the stratum spinosum (superficial layer) of the epidermis; under high magnification these cells have spiky membrane projections (giving them a “prickly” appearance). Images shown are representative of at least 20 biological replicates identified within this study and related studies from the Egan lab. Scale bars in each large panel = 100 μm. Inset images are expanded by 2.4 fold.

Supplementary Figure 6

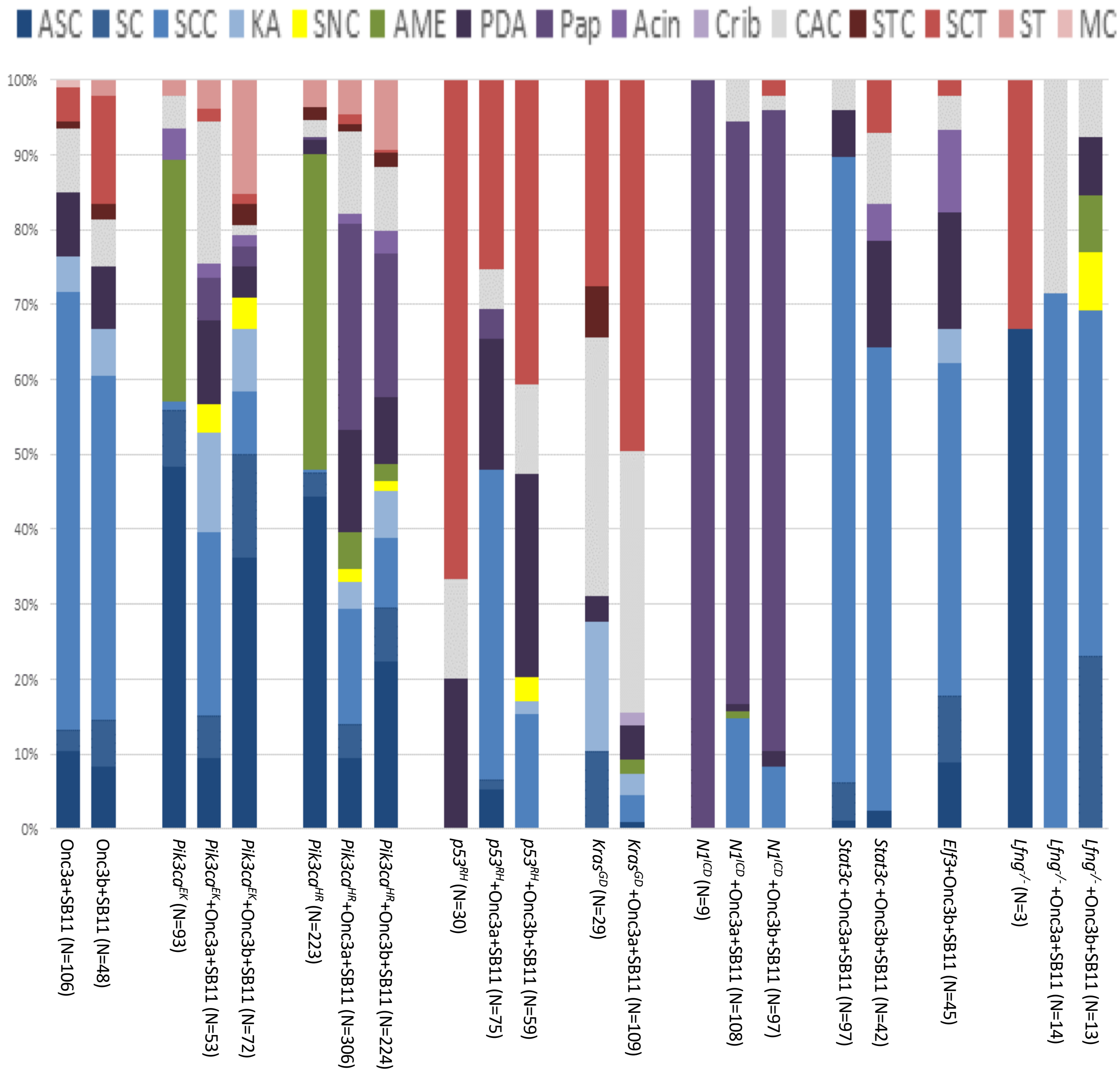

Supplementary Figure 6. Histopathologic classification of SB-induced mammary tumors from each driver-specific cohort. (A) The proportion of tumor types identified in each SB screen are shown. All tumors were categorized based on classification frameworks established by Robert Cardiff and colleagues. ASC-Adenosquamous Carcinoma, SC-Squamous Cyst, SCC-Squamous Cell Carcinoma, KA-Keratoacanthoma, SNC-Solid Nodular Carcinoma, AME-Adenomyoepithelioma, PDA-Poorly Differentiated Adenocarcinoma, Pap-Papillary, Acin-Acinar, Crib-Cribiform, CAC-Complex Adenocarcinoma, STC- Scirrhous Tubular Carcinoma, SCT-Spindle Cell Tumor, ST-Scirrhous Tumor, MC-Metaplastic Carcinoma. Note, oncogene activation or tumor suppressor gene deletion in each cohort was coupled to SB11 transposase expression and T2Onc3 transposon mobilization through expression of Cre recombinase (from MMTV-Cre^{NLST} transgene).

Adenosquamous Carcinoma

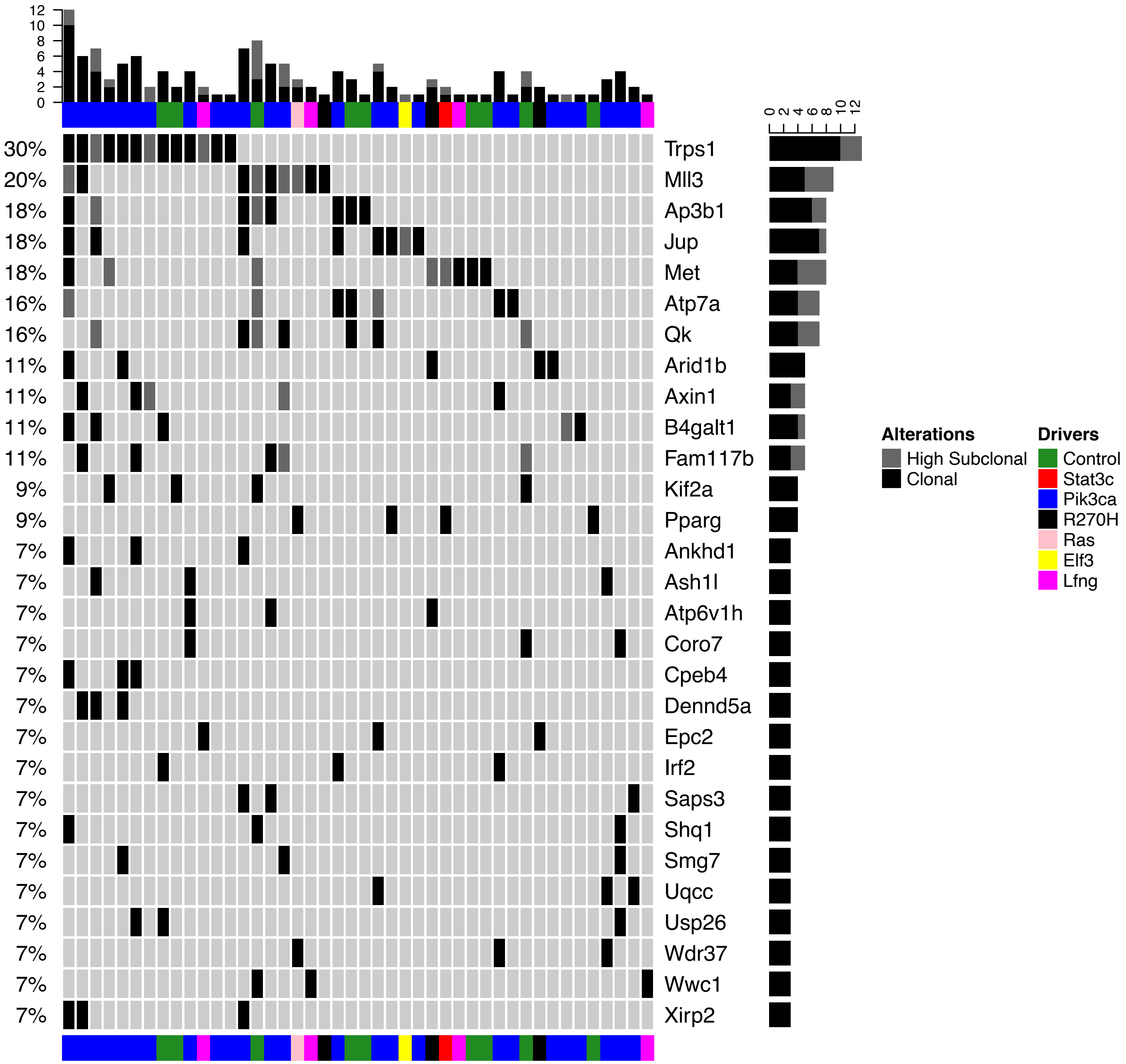

Papillary

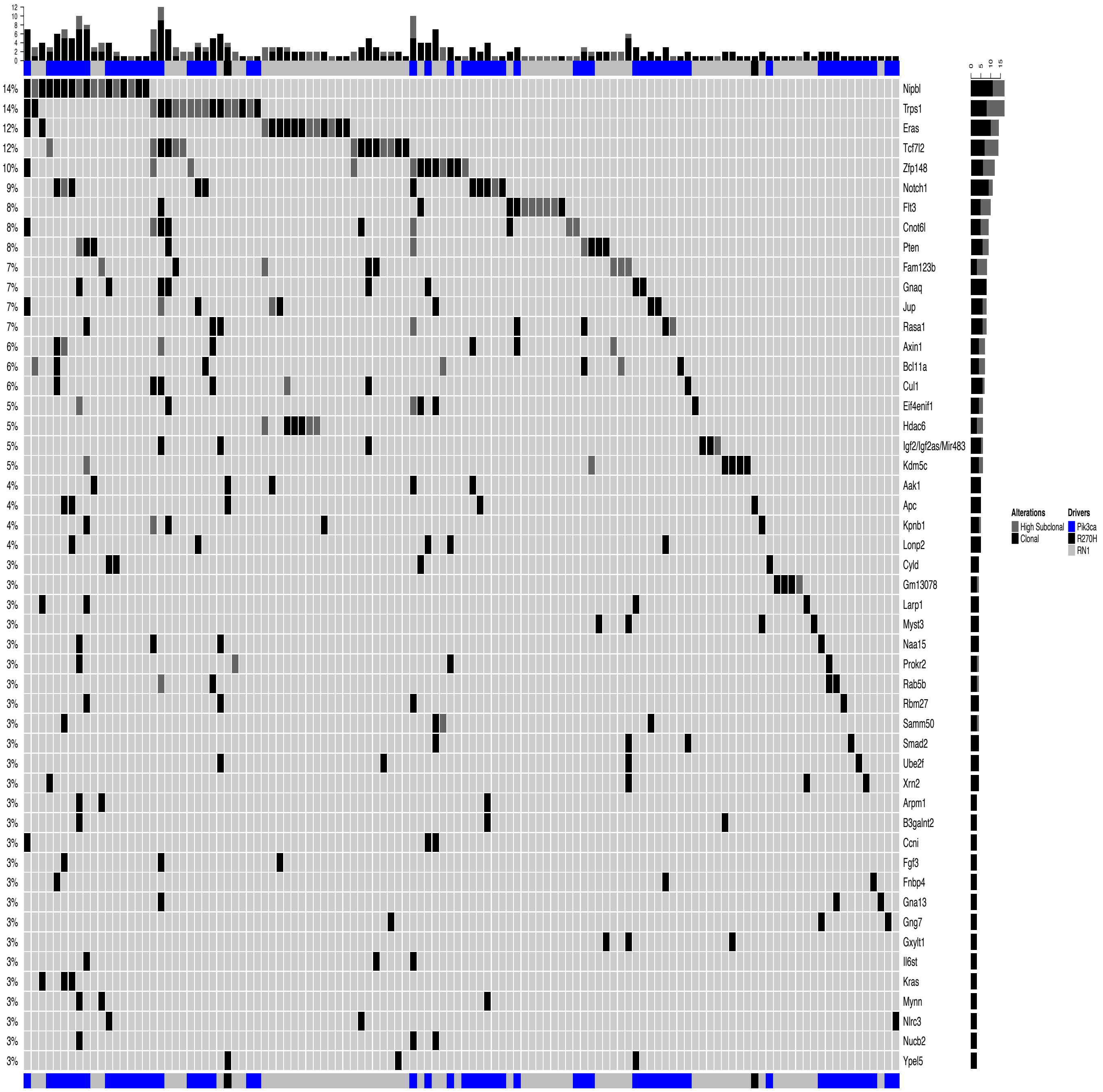
12
10
8
6
4
2
0
0
5
10
15
14% Nipbl
14% Trps1
12% Eras
12% Tcf7l2
10% Zfp148
9% Notch1
8% Flt3
8% Cnot6l
8% Pten
7% Fam123b
7% Gnaq
7% Jup
7% Rasa1
6% Axin1
6% Bcl11a
6% Cul1
5% Eif4enif1
5% Hdac6
5% Igf2/Igf2as/Mir483
5% Kdm5c
4% Aak1
4% Apc
4% Kpnb1
4% Lonp2
3% Cyld
3% Gm13078
3% Larp1
3% Myst3
3% Naa15
3% Prokr2
3% Rab5b
3% Rbm27
3% Samm50
3% Smad2
3% Ube2f
3% Xrn2
3% Arpm1
3% B3galnt2
3% Ccni
3% Fgf3
3% Fnbp4
3% Gna13
3% Gng7
3% Gxylt1
3% Il6st
3% Kras
3% Mynn
3% Nlrc3
3% Nucb2
3% Ypel5
Alterations
High Subclonal
Clonal
Drivers
Pik3ca
R270H
RN1

Poorly Differentiated Adenocarcinoma

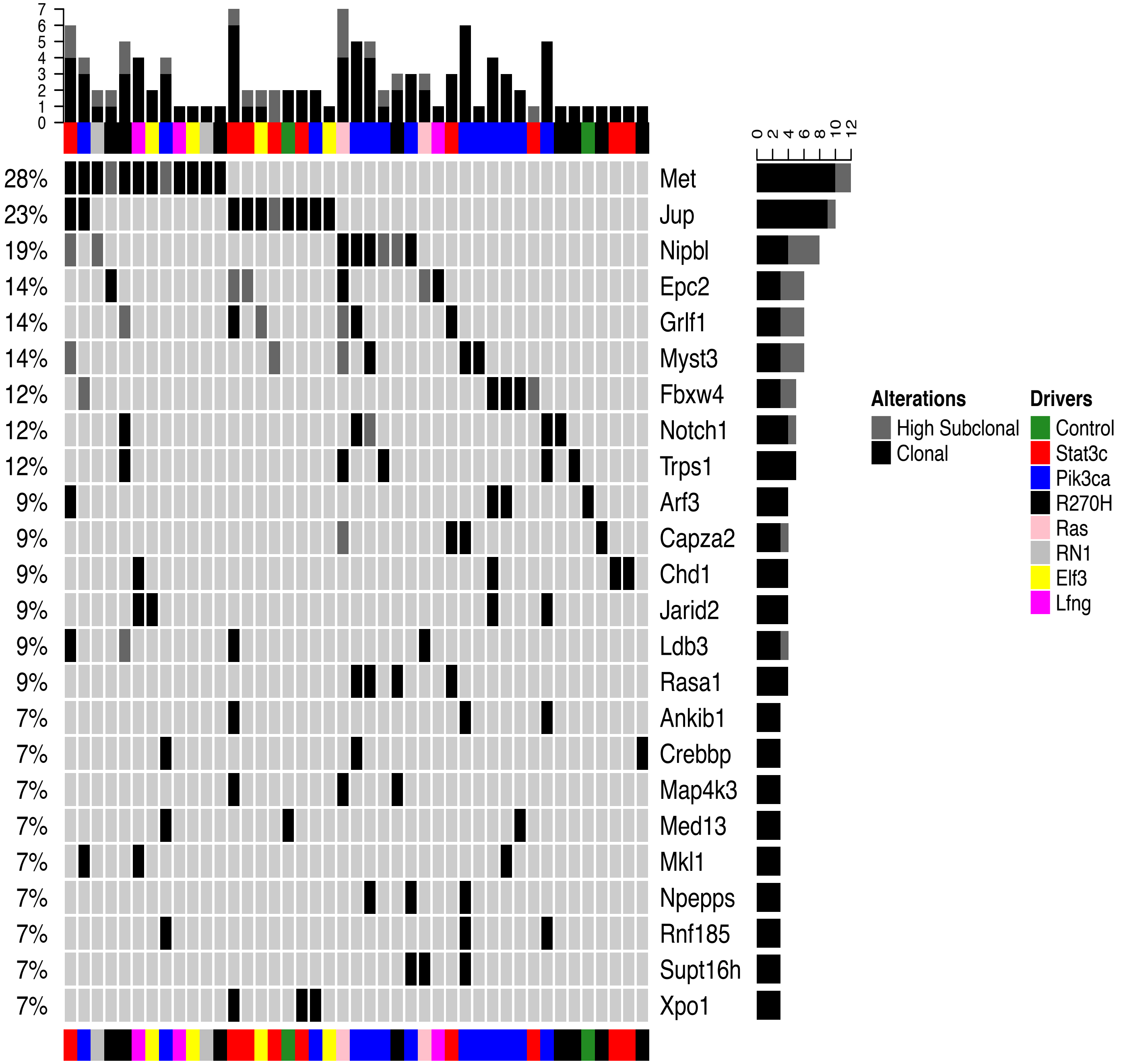

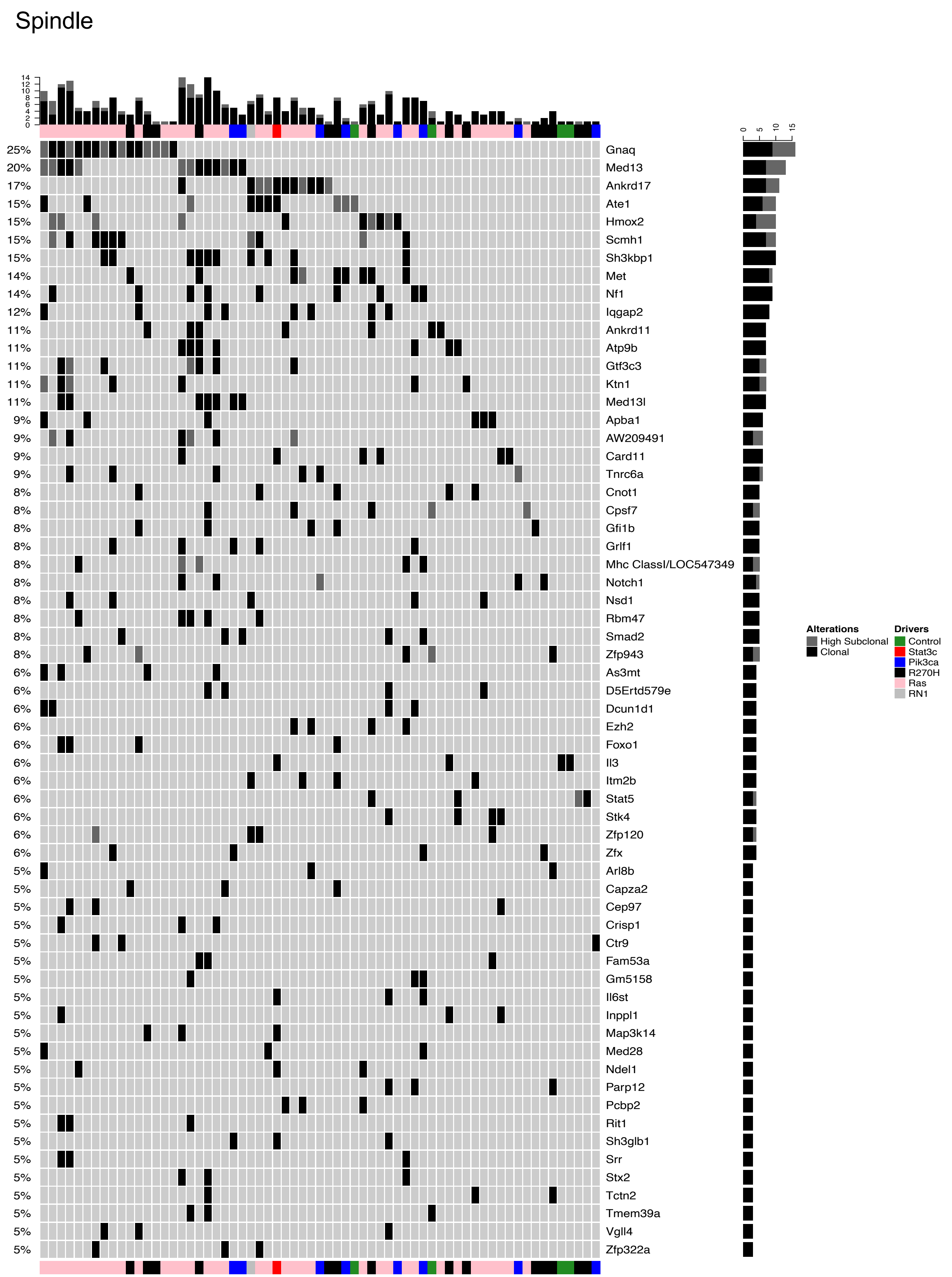

Spindle
25% Gnaq
20% Med13
17% Ankrd17
15% Ate1
15% Hmox2
15% Scmh1
15% Sh3kbp1
14% Met
14% Nf1
12% Iqgap2
11% Ankrd11
11% Atp9b
11% Gtf3c3
11% Ktn1
11% Med13l
9% Apba1
9% AW209491
9% Card11
9% Tnrc6a
8% Cnot1
8% Cpsf7
8% Gfi1b
8% Grlf1
8% Mhc ClassI/LOC547349
8% Notch1
8% Nsd1
8% Rbm47
8% Smad2
8% Zfp943
6% As3mt
6% D5Ertd579e
6% Dcun1d1
6% Ezh2
6% Foxo1
6% Il3
6% Itm2b
6% Stat5
6% Stk4
6% Zfp120
6% Zfx
5% Arl8b
5% Capza2
5% Cep97
5% Crisp1
5% Ctr9
5% Fam53a
5% Gm5158
5% Il6st
5% Inppl1
5% Map3k14
5% Med28
5% Ndel1
5% Parp12
5% Pcbp2
5% Rit1
5% Sh3glb1
5% Srr
5% Stx2
5% Tctn2
5% Tmem39a
5% Vgll4
5% Zfp322a
Alterations
High Subclonal
Clonal
Drivers
Control
Stat3c
Pik3ca
R270H
Ras
RN1

Squamous

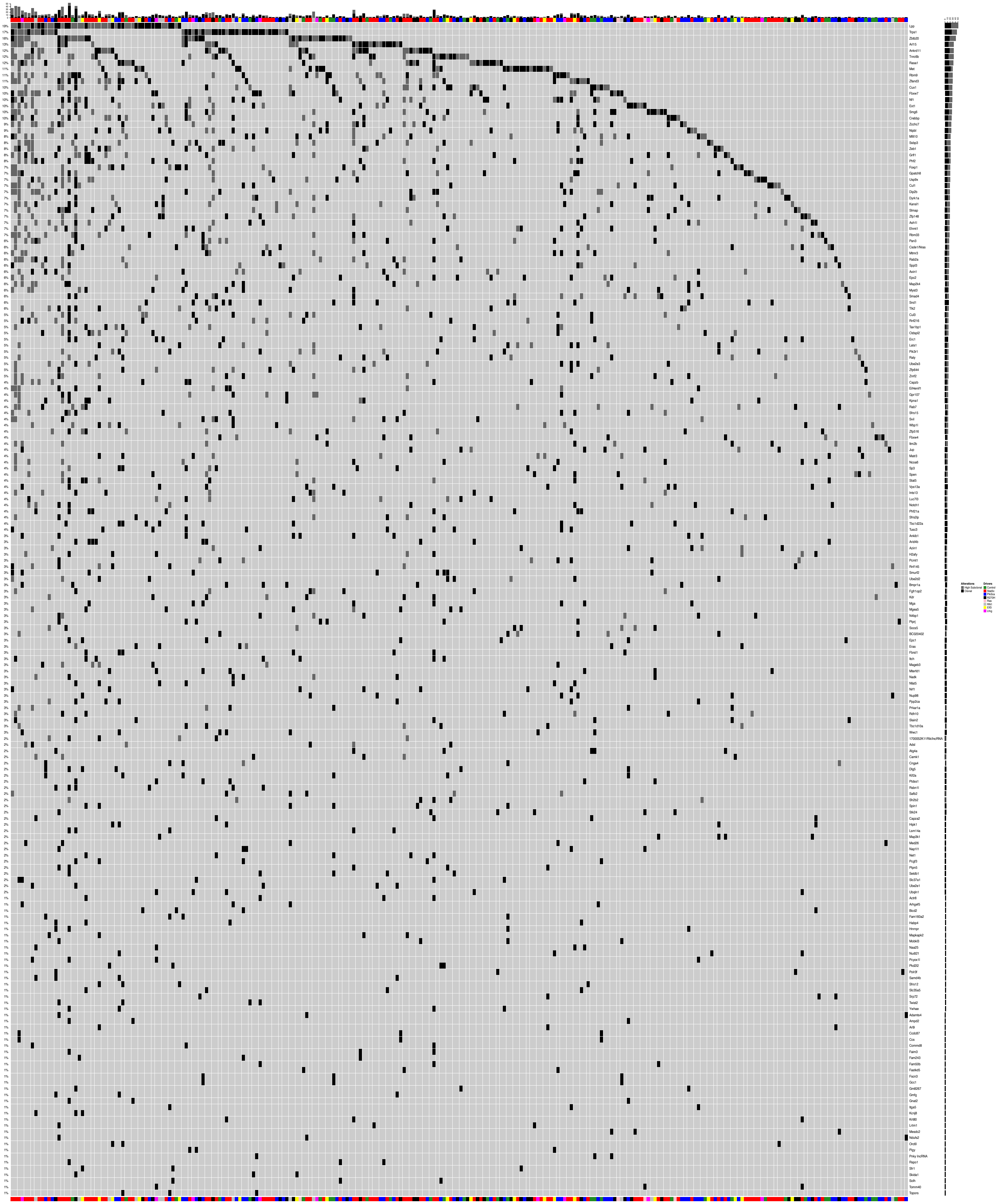

Supplementary Figure 7. Oncoprint analysis shows complete list of clonal gCIS from each histopathology-specific cohort

List of Clonal gCIS (with subclonal tumors added) from each histopathology-specific SB screen. Note, the percentage of tumors with each gene targeted by SB is shown on the y-axis to the left for each bar graph, the number of tumors with clonal vs. subclonal SB targeting by SB is shown on the y-axis to the right, whereas the number of identified gCIS in each tumor is shown on the x-axis above each bar graph. Note, for example, a high-frequency gCIS identified in Adenosquamous tumors was *Trps1*. This gene had SB insertions in 30% of tumors from this cohort (left side y-axis). Also, as shown on the right hand y-axis, clonal insertions were identified in 10/13 tumors from the cohort with insertions in the gene, whereas the remaining 3/13 had subclonal inserts. Finally, the tumor depicted in the most left hand column from the same cohort had 12 different gCIS insertions, 10l of which scored as clonal.

Reagent or Resource	Source	Identifier
**Experimental Models: Organisms/Strains**		
Mouse: MMTV-Cre^{NLST} (*Tg(MMTV-cre)#Tfln*)	[1]	N/A
Mouse: Nf1^{loxP} (*Nf1^{tm1Par}/J*)	[2]	Jax: 017639
Mouse: Notch1^{loxP} (*Notch1^{tm2Rko}*)	[3]	N/A
Mouse: R26$^{LSL-Elf3}$ (*FVB*)	This Work	N/A
Mouse: R26$^{LSL-Notch1(IC\Delta C)}$ (*FVB.Gt(ROSA)26Sor$^{tm1(Notch1)Dam}$/J*)	[4]	Jax: 008159
Mouse: R26$^{LSL-Stat3C}$ (*FVB*)	[5]	N/A
Mouse: R26$^{LSL-SB11}$ (*C57BL/6J.C3H-Gt(ROSA)26Sor$^{tm1(SB11)}$*)	[6]	N/A
Mouse: R26-LSL-*Pik3ca^{H1047R}* (*FVB.129S6-Gt(ROSA)26Sor$^{tm1(Pik3ca*H1047R)Egan}$/J*)	[7]	Jax: 016977
Mouse: R26-LSL-*Pik3ca^{E545K}* (*FVB*)	[8]	N/A
Mouse: R26-LSL-*Pik3ca^{E545K}* (*FVB*)	[7]	N/A
Mouse: R26-LSL-*Pik3ca$^{E545K-H1047R}$* (*FVB*)	This work	N/A
Mouse: T2Onc3a (Tg12740)	[6]	TG12775
Mouse: T2Onc3b (Tg12775)	[6]	TG12740
Mouse: *Trp53$^{LSL-R270H}$* (*B6.129S4-Trp53$^{tm3.1Tyj}$/J*)	[9]	Jax: 008182
Mouse: *Trps1^{dEx4}* (*Trps1$^{tm1.1Shiv}$*)	[10]	N/A
Mouse: *Fbxw7^{loxP}* (*Fbxw7^{tm1Iaai}/J*)	[11]	Jax: 017563
Mouse: *Lfng^{loxP}* (*FVB.129-Lfng$^{tm1.1Egan}$*)	[12]	Jax: 37160
Mouse: *K-Ras^{G12D}* (*B6.129S4-Kras^{tm4Tyj}/J*)	[13]	Jax: 008179
Mouse: *Pten^{loxP}* (*C;129S4-Pten^{tm1Hwu}/J*)	[14]	Jax: 004597
Mouse: *Ctnnb1$^{\delta ex3}$* (*Ctnnb1^{tm1Mmt}*)	[15]	N/A
**Chemicals, Peptides, and Recombinant Proteins**		
High Fidelity (HF) BamHI	New England Biolabs	Cat # R3136
XbaI	New England Biolabs	Cat # R0145S
MluI	New England Biolabs	Cat # R0198S
G418	ThermoFisher Scientific	Cat # 10131027
Phusion Taq	New England Biolabs	Cat # M0530S
10% buffered formalin phosphate	Fisher Scientific	Cat#HC200-20
**Critical Commercial Assays**		
DNeasy Blood and Tissue Kit	Qiagen	Cat # 69506
End-It™ DNA End-Repair Kit	Lucigen Corporation	Cat # ER0720
Fast-Link™ DNA Ligation Kit	Lucigen Corporation	Cat # LK0750H
EndoFree Maxi kit	Qiagen	Cat # 12362
QIAquick PCR Purification Kit	Qiagen	Cat # 28106
RNeasy Kit	Qiagen	Cat # 74104;
QIAshredder	Qiagen	Cat # 79654
Quantitect Reverse Transcription Kit	Qiagen	Cat # 205310
**Western blot Antibodies**		
Rabbit anti-Notch1 mAb	Cell Signaling	D1E11 XP, Cat. #3608
Mouse anti-γ-Catenin mAb	Santa Cruz Biotech.	Cat. # sc-514115

**Deposited Data**		
Genomic library sequence data for all SB tumours	This work	GEO: GSE143503<br>Go to https://urldefense.proofpoint.com/v2/url?u=https-3A__www.ncbi.nlm.nih.gov_geo_query_acc.cgi-3Facc-3DGSE143503&d=DwIBAg&c=Sj806OTFwmuG2UO1EEDr-2uZRzm2EPz39TfVBG2Km-o&r=vId-x21UYvvNICImGuaf7mv5glt9jctbz4aTfS3OKlw&m=DhGRKSDkT9rgD8BMzNm84bo-hDi5KREqVOX1qAZeppE&s=rXVqaU45y8qVfU9YJI2isgZsg4_puOkgLnIlVRzV44c&e=
**Oligonucleotides**		
Primer for genotyping R26-targeted mice (*R26*$^{\text{LSL-Notch1(IC}\Delta\text{C)}}$, *R26*$^{\text{LSL-Stat3C}}$, *R26*$^{\text{LSL-Pik3ca(Mut)}}$): 5'-AAAGTCGCTCTGAGTTGTTAT-3' Common Forward	7	WT: 603 bp<br>R26-targeted: 314bp
Primer for genotyping R26-targeted mice (*R26*$^{\text{LSL-Notch1(IC}\Delta\text{C)}}$, *R26*$^{\text{LSL-Stat3C}}$, *R26*$^{\text{LSL-Pik3ca(Mut)}}$): 5'-GGAGCGGGAGAAATGGATATG-3' WT Reverse.	7	WT: 603 bp<br>R26-targeted: 314bp
Primer for genotyping R26-targeted mice (*R26*$^{\text{LSL-Notch1(IC}\Delta\text{C)}}$, *R26*$^{\text{LSL-Stat3C}}$, *R26*$^{\text{LSL-Pik3ca(Mut)}}$): 5'-GCGAAGAGTTTGTCCTCAACC-3' Mutant Reverse.	7	WT: 603 bp<br>R26-targeted: 314bp
Primer for genotyping MMTV-Cre^{NLST}: 5'-TCGCGATTATCTTCTATATCTTCAG-3' Forward	7	WT: No Band<br>Cre: 420bp
Primer for genotyping MMTV-Cre^{NLST}: 5'-GCTCGACCAGTTTAGTTACCC-3' Reverse	7	WT: No Band<br>Cre: 420bp
Primer for genotyping Nf1$^{\text{tm1Par}}$/J: 5'-ACCTCTCTAGCCTCAGGAATG-3' Wild Type Reverse	2	WT: 450bp<br>loxP: 350bp
Primer for genotyping Nf1$^{\text{tm1Par}}$/J: 5'-CTTCAGACTGATTGTTGTACCTGA-3' Common Forward	2	WT: 450bp<br>loxP: 350bp
Primer for genotyping Nf1$^{\text{tm1Par}}$/J: 5'-TGATTCCCACTTTGTGGTTCTAAG-3' Mutant Reverse	2	WT: 450bp<br>loxP: 350bp

Primer for genotyping Notch1^{tm2Rko}: 5'-CTGACTTAGTAGGGGGAAAAC-3' Forward	3	WT: 300bp<br>Flox: 350bp
Primer for genotyping Notch1^{tm2Rko}: 5'-AGTGGTCCAGGGTGTGAGTGT-3' Reverse	3	WT: 300bp<br>Flox: 350bp
Primer for genotyping C57BL/6J.C3H-Gt(ROSA)26Sor$^{tm1(SB11)}$: 5'-CACTTGCTCTCCCAAAGTCGCT-3' Common Forward	6	WT: 444bp<br>SB11: 342bp
Primer for genotyping C57BL/6J.C3H-Gt(ROSA)26Sor$^{tm1(SB11)}$: 5'-GGGGTGGTGATATAAACTTGAGGCT-3' Mutant Reverse (gene-targeted)	6	WT: 444bp<br>SB11: 342bp
Primer for genotyping C57BL/6J.C3H-Gt(ROSA)26Sor$^{tm1(SB11)}$: 5'-GGCGGATCACAAGCAATAATAACC-3' WT Reverse	6	WT: 444bp<br>SB11: 342bp
Primer for genotyping T2Onc3b: 5'-ATGAGATGGGAGCAGTGGAG-3' Common Forward	6	WT: 105bp<br>OncB: 362bp
Primer for genotyping T2Onc3b: 5'-GCAGCTTTTTCTGGACACCT-3' WT Reverse	6	WT: 105bp<br>OncB: 362bp
Primer for genotyping T2Onc3b: 5'-CTGGTTTATTGCTGGCGTTT-3' Mutant Reverse (T2Onc3)	6	WT: 105bp<br>OncB: 362bp
Primer for genotyping T2Onc3a: 5'-TGCTTACCCATCTCCAACCT-3' Common Forward	6	WT: 451bp<br>OncA: 315bp
Primer for genotyping T2Onc3a: 5'-GTGATGGGAGATGGAAATGG-3' WT Reverse	6	WT: 451bp<br>OncA: 315bp
Primer for genotyping T2Onc3a: 5'-AACTTTATCCGCCTCCATCC-3' Mutant Reverse (T2Onc3)	6	WT: 451bp<br>OncA: 315bp
Primer for genotyping B6.129S4-Trp53$^{tm3.1Tyj}$/J: 5'-AGCTAGCCACCATGGCTTGAGTAAGTCTGCA-3' Mutant Forward	9	WT: 166bp<br>R270H: 270bp
Primer for genotyping B6.129S4-Trp53$^{tm3.1Tyj}$/J: 5'-CTTGGAGACATAGCCACACTG-3' Common Reverse	9	WT: 166bp<br>R270H: 270bp
Primer for genotyping B6.129S4-Trp53$^{tm3.1Tyj}$/J: 5'-TTACACATCCAGCCTCTGTGG-3' WT Forward	9	WT: 166bp<br>R270H: 270bp
Primer for genotyping Trps1$^{tm1.1Shiv}$: 5'-GTTAAGTTATTGCTCTCATTTCGGAC-3' Common Forward	10	Wild type – 1500 bp<br>ΔGT – 850 bp
Primer for genotyping Trps1$^{tm1.1Shiv}$: 5'-ACACCAGATCCTCTTCGCCTC-3' WT Reverse	10	Wild type – 1500 bp<br>ΔGT – 850 bp

Primer for genotyping Trps1$^{tm1.1Shiv}$: 5'-CTTCTATCGCCTTCTTGACG-3' Mutant Reverse	10	Wild type – 1500 bp<br>ΔGT – 850 bp
Primer for genotyping Fbxw7^{tm1Iaai}/J:<br>5'-ATTGATACAAACTGGAGACGAGG-3' Forward	11	Wild Type – 315 bp<br>Mutant – 500 bp
Primer for genotyping Fbxw7^{tm1Iaai}/J:<br>5'-ATAGTAATCCTCCTGCCTTGG C-3' Reverse	11	Wild Type – 315 bp<br>Mutant – 500 bp
Primer for genotyping 129P2-Trp53^{tm3Tyj}/J:<br>5'-TTACACATCCAGCCTCTGTGG-3'<br>WT Forward	9	WT: 371 bp
Primer for genotyping 129P2-Trp53^{tm3Tyj}/J:<br>5'AGCTAGCCACCATGGCTTGAGTAAGTCTGCA-3' LSL-Forward	9	LSL Mut: ~380bp
Primer for genotyping 129P2-Trp53^{tm3Tyj}/J:<br>5'- CTTGGAGACATAGCCACACTG-3' Reverse	9	WT: 371 bp<br>LSL Mut: ~380bp
Primer for genotyping FVB.129-Lfng$^{tm1.1Egan}$:<br>5'-ACCTGTCTGAAGTTGGAGAGTGAG-3' Forward	16	WT: 684bp<br>loxP: 950 bp<br>deleted allele: 436 bp
Primer for genotyping FVB.129-Lfng$^{tm1.1Egan}$:<br>5'-AGCAGTTGGTGAGCACCACATTGC-3' Reverse	16	WT: 684bp<br>loxP: 950 bp<br>deleted allele: 436 bp
Primer for genotyping B6.129S4-Kras^{tm4Tyj}/J:<br>5'-TGTCTTTCCCCAGCACAGT-3' WT Forward	13	WT: 250bp
Primer for genotyping B6.129S4-Kras^{tm4Tyj}/J:<br>5'-GCAGGTCGAGGGACCTAATA-3' Mutant Forward	13	Mut: 100bp
Primer for genotyping B6.129S4-Kras^{tm4Tyj}/J:<br>5'-CTGCATAGTACGCTATACCCTGT-3'<br>Common Reverse	13	WT: 250bp<br>Mut: 100bp
Primers for genotyping Ctnnb1^{tm1Mmt}: 5'-AGGGTACCTGAAGCTCAGCG-3' WT Forward	15	Wild Type – 412 bp<br>Mutant – 645 bp
Primers for genotyping Ctnnb1^{tm1Mmt}: 5'-+CAGTGGCTGACAGCAGCTTT-3' WT Reverse	15	Wild Type – 412 bp<br>Mutant – 645 bp
Linker+ Primer Sequence: 5'-GTAATACGACTCACTATAGGGCTCCGCTTAAGGGAC-3'	6	N/A
Linker- Primer Sequence: 5'-Phos-GTCCCTTAAGCGGAG-C3spacer-3'	6	N/A
Barcoded SB primer sequence: 5'-AATGATACGGCGACCACCGAGATCTACACTCTTTCCCTACACGACGCTCTTCCGATCT(barcode)TGTATGTAAACTTCCGACTTCAACTG-3'	6	N/A
Linker-A2 Primer Sequence: 5'-CAAGCAGAAGACGGCATACGAGATCGGTCTCGGCATTCCTGCTGAACCGCTCTTCCGATCTTAGGGCTCCGCTTAAGGGAC-3'	6	N/A

Primer to identify mESC with correctly-targeted Elf3 at the Rosa26 locus: 5'-CGCCTAAAGAAGAGGCTGTG-3' Forward	This work	WT: No Band<br>Elf3: 500bp
Primer to identify mESC with correctly-targeted Elf3 at the Rosa26 locus: 5'-GAAAGACCGCGAAGAGTTTG-3' Reverse	This work	WT: No Band<br>Elf3: 500bp
Primer used to amplify Elf3 cDNA: 5'-CTCAAGCTTGCCACCATGGACTACAAGGACGACGACGATAAGATGGCTGCCACCTGTGAGA-3'	This work	1167bp
Primer used to amplify Elf3 from cDNA: 5'-CGCTCTAGATTAATTCCGACTCTCTCCAACCTC -3' Reverse	This work	1167bp
IRR for SB Primary PCR: 5'-GGATTAAATGTCAGGAATTGTGAAAA-3'	[6]	
IRL for SB Primary PCR: 5'-5'-AAATTTGTGGAGTAGTTGAAAAACGA-3'	[6]	
Linker-A1 for SB primary PCR: 5'-GTAATACGACTCACTATAGGGC-3'	[6]	
**Recombinant DNA**		
pRosa26PAm1	Addgene	Plasmid# 15036
pBGT (pBigT-IRES-GFP) Shuttle Vector	Addgene	Plasmid # #15037
**Software and Algorithms**		
gProfiler (version 0.6.8)	[17]	https://biit.cs.ut.ee/gprofiler/
Cytoscape (version 3.7.1)	[18]	http://www.cytoscape.org/
R Studio	https://www.rstudio.com/	N/A
Cutadapt (v1.8) https://cutadapt.readthedocs.io/en/stable/	[19]	https://cutadapt.readthedocs.io/en/stable/
Novoalign (v3.05.01)	http://www.novocraft.com/products/novoalign/	N/A
**Experimental Models: Cell Lines**		
W4 mouse embryonic stem cells (mESC)	The Center for Phenogenomics (Toronto)	

Supplementary Table 1: Reagents and Resources

Source information on mouse strains used in this study, as well as chemical, enzyme and antibody reagents are provided. Oligonucleotide primer sequences for genotyping mice, software packages used for data analysis and details on ES cells for generation of novel mouse strains are listed.

Supplementary References:

1. Li, G. *et al.* Conditional loss of PTEN leads to precocious development and neoplasia in the mammary gland. *Development* **129**, 4159-70 (2002).
2. Zhu, Y. *et al.* Ablation of NF1 function in neurons induces abnormal development of cerebral cortex and reactive gliosis in the brain. *Genes Dev* **15**, 859-76 (2001).
3. Radtke, F. *et al.* Deficient T cell fate specification in mice with an induced inactivation of Notch1. *Immunity* **10**, 547-58 (1999).
4. Murtaugh, L.C., Stanger, B.Z., Kwan, K.M. & Melton, D.A. Notch signaling controls multiple steps of pancreatic differentiation. *Proc Natl Acad Sci U S A* **100**, 14920-5 (2003).
5. Mesaros, A. *et al.* Activation of Stat3 signaling in AgRP neurons promotes locomotor activity. *Cell Metab* **7**, 236-48 (2008).
6. Dupuy, A.J. *et al.* A modified sleeping beauty transposon system that can be used to model a wide variety of human cancers in mice. *Cancer Res* **69**, 8150-6 (2009).
7. Adams, J.R. *et al.* Cooperation between Pik3ca and p53 Mutations in Mouse Mammary Tumor Formation. *Cancer Res* **71**, 2706-2717 (2011).
8. An, Y. *et al.* Cdh1 and Pik3ca Mutations Cooperate to Induce Immune-Related Invasive Lobular Carcinoma of the Breast. *Cell Rep* **25**, 702-714 e6 (2018).
9. Olive, K.P. *et al.* Mutant p53 gain of function in two mouse models of Li-Fraumeni syndrome. *Cell* **119**, 847-60 (2004).
10. Malik, T.H., Von Stechow, D., Bronson, R.T. & Shivdasani, R.A. Deletion of the GATA domain of TRPS1 causes an absence of facial hair and provides new insights into the bone disorder in inherited tricho-rhino-phalangeal syndromes. *Mol Cell Biol* **22**, 8592-600 (2002).
11. Thompson, B.J. *et al.* Control of hematopoietic stem cell quiescence by the E3 ubiquitin ligase Fbw7. *J Exp Med* **205**, 1395-408 (2008).
12. Xu, K. *et al.* Lunatic fringe deficiency cooperates with the Met/Caveolin gene amplicon to induce basal-like breast cancer. *Cancer Cell* **21**, 626-41 (2012).
13. Jackson, E.L. *et al.* Analysis of lung tumor initiation and progression using conditional expression of oncogenic K-ras. *Genes Dev* **15**, 3243-8 (2001).
14. Groszer, M. *et al.* Negative regulation of neural stem/progenitor cell proliferation by the Pten tumor suppressor gene in vivo. *Science* **294**, 2186-9 (2001).
15. Harada, N. *et al.* Intestinal polyposis in mice with a dominant stable mutation of the beta-catenin gene. *EMBO J* **18**, 5931-42 (1999).
16. Xu, K. *et al.* Lunatic Fringe-mediated Notch signaling is required for lung alveogenesis. *Am J Physiol Lung Cell Mol Physiol* **298**, L45-56 (2010).
17. Reimand, J. *et al.* Pathway enrichment analysis and visualization of omics data using g:Profiler, GSEA, Cytoscape and EnrichmentMap. *Nat Protoc* **14**, 482-517 (2019).
18. Shannon, P. *et al.* Cytoscape: a software environment for integrated models of biomolecular interaction networks. *Genome Res* **13**, 2498-504 (2003).
19. Martin, M. Cutadapt removes adapter sequences from high-throughput sequencing reads. *Embnet. J.*, 10-12 (2011).
