# Supplementary material for: Single allele loss-of-function mutations select and sculpt conditional cooperative networks in breast cancer
Source: Nat Commun. 2021 Sep 2;12:5238. doi: 10.1038/s41467-021-25467-w (PMC8413298; doi:10.1038/s41467-021-25467-w)
Supplement: Supplementary file 3 — Description of Additional Supplementary Files [file 41467_2021_25467_MOESM3_ESM.pdf]

Description of Additional Supplementary Files

File Name: Supplementary Data 1
Description: **Clonal and subclonal gCIS for all cohorts:** Clonal and high-subclonal gCIS are listed together with p-value, q-value and number of tumors within a cohort with SB insertion into each gene of interest. Data on the following cohorts are provided as separate tabs: Control Cohort, *Pik3ca*^{E545K}, *Pik3ca*^{H1047R}, *p53*^{R270H}, *K-Ras*^{G12D}, *Notch1*^{ICD}, *Stat3C*, *Elf3*, *LFng*$^{loxP/loxP}$, Combined Cohort, Adenosquamous Carcinomas, Papillary Tumors, Poorly Differentiated Adenocarcinomas, Spindle Tumors and Squamous Tumors. p-values are based on a one-sided Chi-Squared test, k=1 and q-value represent the Bonferroni corrected p-value (see reference 21, Brett et al., 2011).

File Name: Supplementary Data 2
Description: **Data on clonal and subclonal gCIS linked to tumor identity for analysis of gCIS co-selection.** Data on the following cohorts are provided as separate tabs: Control Cohort, *Pik3ca*^{E545K}, *Pik3ca*^{H1047R}, *p53*^{R270H}, *K-Ras*^{G12D}, *Notch1*^{ICD}, *Stat3C*, *Elf3*, *LFng*$^{loxP/loxP}$, Combined Cohort, Adenosquamous Carcinomas, Papillary Tumors, Poorly Differentiated Adenocarcinomas, Spindle Tumors and Squamous Tumors. Part of this data is presented in Oncoprint form as Figures 1 and 4, as well as in more complete form as Supplementary Figures 3 and 7.

File Name: Supplementary Data 3
Description: **Driver-specific, non-specific and histology specific gCIS lists.** Shown in separate tabs are i) Combined Clonal data: A comparison of clonal gCIS data from all driver-specific lists combined after cohort-specific analysis vs. the list of clonal gCIS obtained from analyzing Control, *Pik3ca*^{E545K}, *Pik3ca*^{H1047R}, *p53*^{R270H}, *K-Ras*^{G12D}, *Notch1*^{ICD}, *Stat3C* and *Elf3* mice as one large cohort, ii) Combined Pathology data: List of gCIS obtained when data from all histology based cohort analysis is combined, and iii) Combined gCIS lists: Lists are presented for all clonal and subclonal gCIS (Driver-specific, driver non-specific and pathology-specific lists added together in each case). Also shown is the total list of (1089) gCIS identified in our study.

File Name: Supplementary Data 4
Description: **Bioinformatic data shows gCIS responsible for identification of specific pathways or biological processes highlighted in Figures 2, 3 and 5.** One-sided ranked hypergeometric tests were used to identify pathways and processes effected by gCIS identified in each cohort. In each case, the genes underlying each designated altered pathway or process are listed, as are the p-values associated with each identified node.

File Name: Supplementary Data 5
Description: **Data on histology for each tumor.** The mammary gland involved, as well as tumor histology is listed for lesions in each driver-specific cohort.

File Name: Supplementary Data 6
Description: **Map positions for human orthologues of gCIS.** Orthologues of gCIS identified in the current screen as well as those from SB screens from multiple tumor types are analyzed with respect to chromosome map position. Tabs for the following data are shown - Our MMTV Sleeping Beauty gCIS: Human orthologues for gCIS identified in the current screen are listed with their chromosomal map position, Possible alternative drivers: A list of potential alternative drivers are listed for gCIS identified in our screen that map near each other (less than 5 kbp apart), Our Mouse-specific gCIS: Identification of mouse gCIS from our screen for which a single human orthologue is not obvious, some of these are found to sit beside genes that may well be the real target for an SB-mediated tumor-promoting alteration (highlighted in yellow), Other Mammary SB screens: Human orthologues for gCIS identified in other mammary-specific SB screens are listed with their chromosomal map position, gCIS from 19 SB screens: Human orthologues for gCIS identified in SB screens from multiple tumor types are analyzed with respect to chromosome map position, and Candidate hTSG-Chr 8p, 16q, 17p: Highlighted are gCIS identified in the current study, other mammary-specific SB screens or in non-mammary SB screens that map to chromosome arms showing high-frequency hemizygous loss in human breast cancer, as well as in other tumor types (focusing on genes mapping to 8p, 16q and 17p). These gene lists are likely to include many haploinsufficient tumor suppressors driving tumor formation and/or progression.

File Name: Supplementary Data 7
Description: **gCIS data from this study are compared to data from other functional genomic screens for cancer genes in the mouse mammary gland, and to the list of focally altered breast cancer genes as well as to the pan-cancer gene list identified in human tumors.** Putative driver genes identified in human and mouse breast/mammary tumors are presented in tabs. Other Mammary SB data (Clonal): Sleeping Beauty cancer gene discovery screens in the mouse mammary gland are listed, Single-copy SB screen PTEN mut: SB target genes identified in mammary tumors from PTEN conditional mutant mice, Human BC and Pan-Cancer genes: A list of genes showing recurrent focal mutation or copy number change in human breast cancer (99 genes) or in a pan-cancer screen (299 genes), MMTV viral insertion sites: Genes identified as MMTV common insertional sites in mouse mammary tumors, Our MMTV-Cre SB Screens-Clonal: Clonal gCIS identified in mammary tumors as described in the current study, Our MMTVCre SB Screen Subclonal: Subclonal gCIS identified in mammary tumors as described in the current study, All Mammary SB Screen Data: A comparison of putative driver genes identified in the current study with SB targets from other mammary tumor screens as well as tumor-associated MMTV-insertion sites, and gCIS overlap with BC genes: Relationship between gCIS identified in the current study with 99 human focally mutated breast cancer genes & 299 human pan-cancer genes.

File Name: Supplementary Data 8
Description: **Nanostring Codeset data.** Shown are coordinates for gene probes used in Nanostring analysis as described in Figure 7.
